# Fundamental Study of Density Functional Theory Applied to Triplet State Reactivity: Introduction of the TRIP50 Data Set

**DOI:** 10.1021/acs.jctc.6c00144

**Published:** 2026-03-19

**Authors:** William B. Hughes, Mihai V. Popescu, Robert S. Paton

**Affiliations:** Department of Chemistry, 224023 Colorado State University, Ft. Collins, Colorado 80523-1872, United States

## Abstract

The recent development of organic visible-light active photosensitizers has enabled the development of many novel triplet transformations, the mechanistic studies of which often rely on computation due to the short lifetime of the excited state intermediates. However, in contrast to studies of ground state reactivity, there has been little discussion of the best practices when using density functional theory to model triplet state reactions. Here, we report the first benchmark of density functionals on triplet reaction mechanisms. Barrier heights and thermodynamic values were computed for a set of 50 organic reactions using 45 functionals, with reference values obtained using high-level DLPNO–CCSD­(T) calculations extrapolated to the complete basis set limit. In the course of this study, we observed a common tendency for triplet SCF calculations to converge non-*Aufbau* solutions, resulting in catastrophic predictions in both thermochemistry and activation energy barriers and leading to errors as high as 26.4 kcal/mol. Modifications to the initial SCF guess are proposed as a solution to such errors, enabling accurate comparison of functional performance. Range-separated hybrid functionals were found to consistently outperform their non-range-separated versions, while rungs below hybrid meta-GGA produce high errors compared to reference values. We recommend the best-performing single hybrid functionals ωM06, ωB97M, M06–2X, and M05–2X for their balance of high accuracy and computational efficiency.

## Graphical abstract

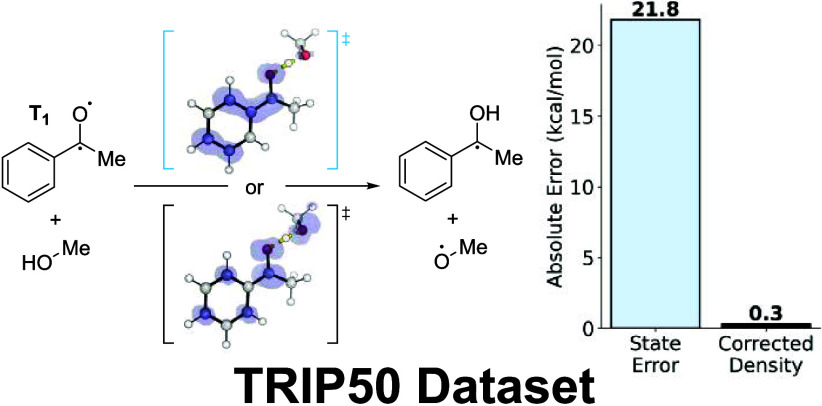

## Introduction

The discovery and development of organic reactions occurring in the triplet excited state has allowed for transformations unique from those enabled via ground state reactivity, opening up new synthetic pathways for chemists to exploit.^1,2^ In particular, the recent development of organic^3^ visible-light active^4^ photosensitizers for use as Dexter energy transfer catalysts^5,6^ has led to a dramatic increase in publications on the topic (Figure 1). This has led to a concomitant increase in the study of reaction mechanisms that proceed through the triplet state.

**Figure 1:**
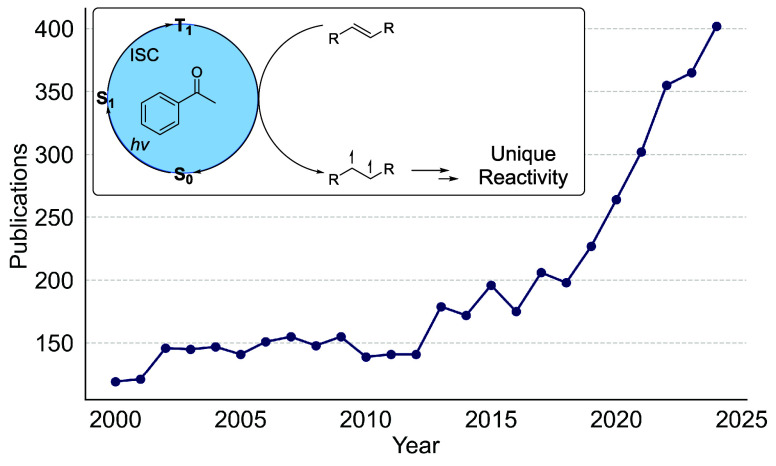
Annual publications featuring “Triplet Energy Transfer” in the title or abstract (SciFinder search). Inset: Representative triplet energy transfer photocatalytic cycle illustrating Dexter energy transfer and associated unique reactivity.

However, probing these reaction pathways is challenging due to the scarcity of methods that provide direct evidence for a triplet mechanism. Experimental techniques for mechanistic studies of proposed triplet state reactions are primarily limited to quenching experiments, such as Stern–Volmer analysis,^7^ and time-resolved spectroscopic techniques, such as transient absorption spectroscopy^8^ and time-resolved photoluminescence spectroscopy (TRPL),^9^ with only the latter able to provide kinetic information regarding triplet elementary steps. As such, computational quantum chemical studies have emerged as an important partner to experimental mechanistic studies when determining the operative mechanism for an excited state reaction. In particular, Kohn–Sham Density Functional Theory^10,11^ (KS-DFT) has emerged as a widely used methodology, as practitioners attempt to balance accuracy with computational cost. These studies target multistep reaction energy profiles and in-depth analysis of relevant intermediate and transition structures in the triplet state.

While evaluation of triplet mechanisms via DFT remains prevalent in the literature,^12–23^ there has been little to no discussion of best practices for using DFT as a tool to probe the triplet excited state (in contrast to singlet–triplet energy gaps). Moreover, the “democratization” of computational chemistry due to the advent of improved computational capabilities, more robust algorithms, and automated workflows has led many experimental chemists to now employ computational tools as an essential tool in their research. As such, the lack of best practice recommendations can lead to the treatment of DFT as a “black box” without proper consideration of the quantum chemical model being used. Additionally, the majority of DFT methods are not evaluated or parametrized using substantial triplet reaction data during the development process. Indeed, the most comprehensive quantum chemical data set used for the quantitative assessment of density functional approximations, the GMTKN55 data set,^24^ does not explicitly consider any triplet reaction barrier heights or thermochemistry. This raises the question of whether prior assessments of functional accuracy are transferable to studies in the triplet state. Due to the rapid expansion of newly developed triplet-state reactions, and the lack of information on the reliability of modern density functionals to this chemistry (along with specific recommendations), there is a pressing need for a quantitative assessment of density functionals across a range of triplet reaction mechanisms.

Herein, we report the first detailed assessment of density functionals on triplet reaction mechanisms, containing thermodynamic and kinetic values for a novel set of 50 representative elementary reaction steps proposed to proceed through a triplet transition state. The resulting TRIP50 data set, split into seven commonly reported reaction types, was used in a benchmark of density functional approximations to determine their efficacy in describing triplet state reactivity. During the analysis of the results, it was observed that the default implementation DFT in common quantum chemical packages can lead to non-*Aufbau* solutions to the final computed electron density. These errors result in overall catastrophic predictions for thermochemical values, indicating that a “black box” approach toward triplet state DFT calculations can lead to erroneous predictions. Potential solutions to these “state errors” and best practices for modeling triplet state reactivity with DFT are discussed. While there are general similarities in terms of how functionals at different rungs on Jacob’s ladder perform in this task and against the GTMKN55 data set,^24^ we highlight the importance of range separation and the limited applicability of hybrid-, meta-, and “pure” generalized gradient approximation (GGA) functionals in accurately describing triplet reactivity. The functionals that best balance performance with efficiency were found to be M05–2X, M06–2X, ωM06, and the ωB97 family of functionals.

## Results and Discussion

### Methodology and Data Set Selection

In recent decades, KS-DFT has become the *de facto* “workhorse” method of the majority of quantum mechanical structure investigations and mechanistic studies. Along with this rise in usage, an increasing interest in benchmarking of these methods has emerged, both for accuracy describing general main group chemistry^24,25^ and for specific areas of chemical space.^26–32^ Owing to its relative ease of use and favorable cost-accuracy balance, KS-DFT can often be treated as a “black box” and assumed to consistently optimize geometries and electronic wave functions to the desired state. This can especially be seen in large scale, high-throughput studies in which the scale of the data generated is too large for the manual scrutiny of each structure.^33–35^

While in recent years there have been numerous technical advancements in DFT-based studies of excited states,^36–38^ studies of such systems have been traditionally limited to unrestricted Kohn–Sham (UKS) and restricted open-shell Kohn–Sham (ROKS) DFT or to time-dependent DFT (TD-DFT). The latter is typically used for the determination of excitation energies and calculations for photoabsorption spectra.^39^ However, it has become evident that TD-DFT has numerous drawbacks and can often lead to qualitatively incorrect results for excited states.^40–45^

In the study of excited states, UKS- and ROKS-DFT are typically considered to be limited to evaluations of only the first triplet excited state.^46^ Fortunately, as Kasha’s rule would suggest, the rate of internal conversion to the lowest energy state for a given multiplicity is typically much faster than the rate of reactive steps. Therefore, reactivity in the triplet state for the majority of reactions is expected to occur in the T_1_ excited state, allowing KS-DFT to be implemented in these cases. Thus, a large proportion of computational studies of triplet reaction mechanisms utilize KS-DFT.

To develop a data set of reactions, literature examples of reactions studied experimentally or computationally for which the operative mechanism is thought to proceed through at least one triplet transition state were considered. Reactions were selected to create a diverse data set including transformations involving different forming/breaking bonds (C–C, C–O, C–S, C–Hal, Si–X, N–X) along with H–atom Transfer (HAT) reactions. Within the same reaction type, we also include structures with varying steric and electronic effects. The size of these systems (ranging from 4 to 20 heavy atoms across the data set) was chosen to be tractable at the reference level of theory (Figure 2). Systems that were too large to model efficiently were truncated to preserve the electronic and steric environment around the reactive atoms. For reactions for which the exact order of mechanistic steps is unknown, such as in the case of stepwise photochemical cycloadditions, each possible excited state elementary step was evaluated. In total, 50 elementary reaction steps were chosen to be modeled.^47–76^

**Figure 2:**
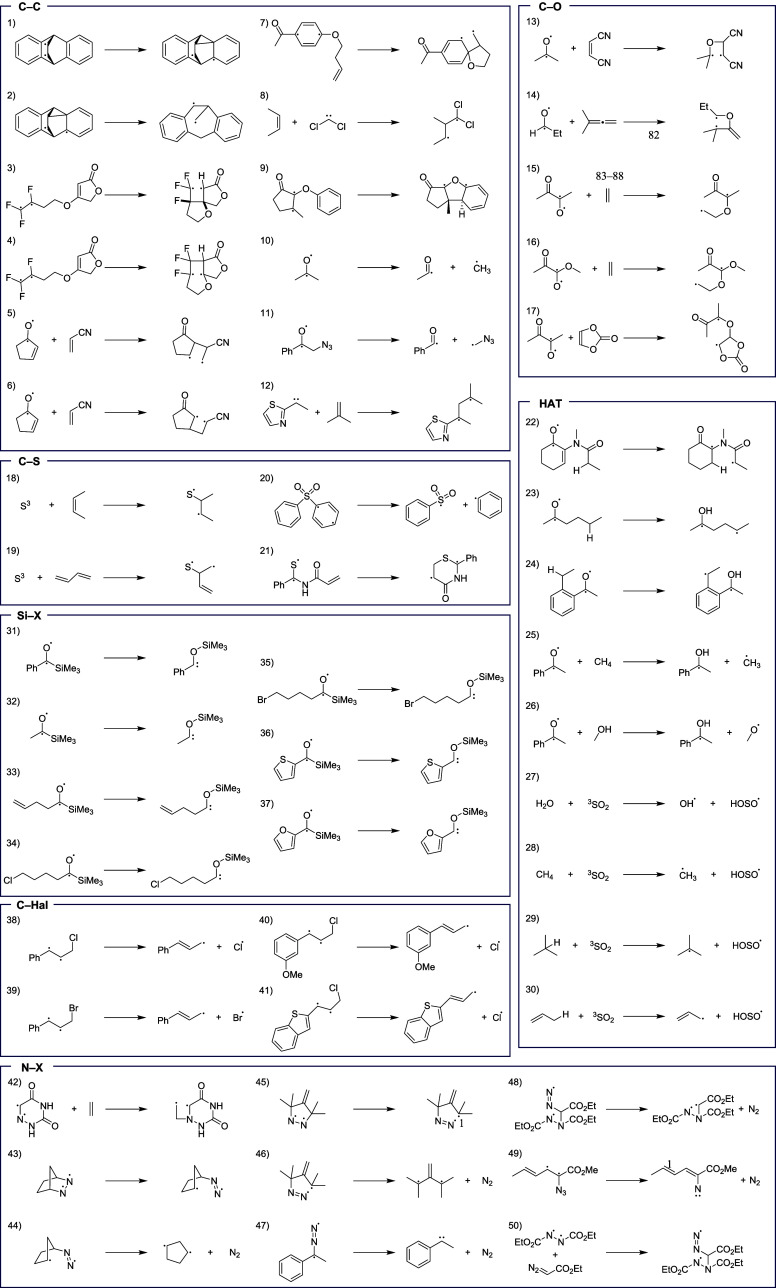
Set of 50 representative triplet state reactions over seven categories: C–C, C–O, C–S, HAT, Si–X, C–Hal, and N–X.

Several stepwise cycloadditions involving a C–C, C–O, C–N, and C–S bond formation were investigated, including those involving electron rich and electron neutral substrates. Addition reactions of triplet carbenes and triplet atomic sulfur were also considered, with representative reactions included in the data set. Fragmentation reactions including C–C, C–N, and C–Halogen (C–Hal) homolytic cleavage were included in the data set. Finally, reactions producing triplet carbenes, including the loss of molecular nitrogen and a set of silane migration in acylsilanes, were considered and included in the data set.

In total, this data set represents a diverse set of chemical reactions relevant to various fields of chemistry. This includes reactions relevant for medicinal and synthetic chemists, such as the Zimmerman di-π-methane rearrangement (**1, 2**), Norrish Type I and II reactions (**10, 11, 23, 24**), stepwise [2 + 2] cycloadditions (**3–7, 9, 13–17, 42**), with a subset of those being Paterno-Büchi reactions, Shultz-type 6π heterocyclizations (**9**), and reactions resulting in the evolution of molecular nitrogen from azo and diazo compounds that yield reactive carbon-centered radicals and triplet carbenes (**43–49**). A subset of the HAT and C–S reactions contain triplet sulfur dioxide and atomic sulfur, respectively (**18, 19, 27–30**). These species are common air pollutants whose triplet reactions are of relevance to atmospheric chemistry. Other reactions in the data set are relevant for catalysis (**8**), as well as biological (**42**) and materials (**20**) chemistry.

All geometry optimizations and vibrational frequency calculations were performed in the Gaussian 16 ^77^ software package using the ωB97X-D^78^/def2-TZVP^79^ level of theory. Transition structures were optimized without constraints at the same level of theory as minima using the Berny optimizer using GEDIIS in redundant internal coordinates, as implemented in the Gaussian 16 software package. Convergence criteria for these calculations was set to “tight” and an “ultrafine” grid with 99 radial shells and 590 angular points was selected. Harmonic vibrational frequency calculations at the same level of theory were used to confirm the nature of the stationary points. All minima possess zero imaginary frequencies whereas transition structures possess exactly one imaginary frequency. Conformational sampling was performed for all structures using a combination of RDKit^80^ generated geometries and manual conformation sampling. All conformers were optimized using the optimization level of theory, and the lowest energy conformer of each structure was selected for further analysis. For evaluation, we selected 45 representative functionals spanning GGA, meta-GGA (mGGA), hybrid GGA (HGGA), hybrid meta-GGA (HmGGA), range-separated hybrid (RSH), and double hybrid (DH) functional classes, with the addition of Hartree–Fock (HF) calculations (see Table S1 for details). Single point energy corrections were performed in both the ORCA 6.0.0 ^81^ and Q-Chem 6.0.2 ^82^ software packages using the def2-QZVPP^79^ basis set. Reference values were computed using DLPNO–CCSD­(T)^83–88^ (domain-based local pair natural orbital coupled cluster with singles, doubles, and perturbative triples) using the ORCA 6.0.0 software package. Calculations were performed with TightPNO thresholds, and a two-point complete basis set (CBS) extrapolation scheme was employed using the def2-TZVPP and def2-QZVPP basis sets using the default implementation in the ORCA 6.0.0 software package.

For each reaction considered, the thermochemistry and activation barrier were computed and compared against DLPNO–CCSD­(T) reference values. Reactants and products were defined according to the direction of the productive reaction reported in the original experimental studies. Thermodynamic values were defined as the difference in product and reactant energies and thus could be either positive or negative. For kinetic values, both forward and reverse barrier heights were computed, and the overall error for each functional and reaction was determined by averaging the absolute errors of the forward and reverse barrier heights. Data were collected on each functional for each reaction, and the mean absolute error (MAE) for both barrier height and thermodynamic values was computed for each functional for the overall data set, as well as each subset of reaction types. All energetic values are reported as the computed dispersion-corrected electronic energy, with no further corrections applied to enable ease of comparison of different quantum chemical methods.

### State-Based Error Discovery and Correction

In self-consistent field (SCF) methods, such as KS-DFT, the total electronic energy is minimized subject to orbital orthogonality, starting from an initial guess and then iteratively solving the SCF equations until convergence. For closed-shell organic molecules, this approach tends to reliably yield the lowest energy wave function; this is widely assumed to hold true for T_1_ and S_0_ states.^46^ However, upon convergence, a local (rather than global) minimum of the SCF Lagrangian can be obtained, and often such “non-*Aufbau* solutions” are actually saddle points rather than minima of the total energy.^89,90^ It is known that there exist multiple valid solutions to the SCF equations for most systems, though local, rather than global, minimum solutions are typically obtained only for exotic chemical species.^91,92^ Herein, we report that multiple solutions to the SCF equations exist in the triplet state for a variety of the species studied, and that non-*Aufbau* solutions arise relatively frequently for triplet ground and transition state structures for such common synthetic species as acetophenone, yielding densities corresponding to a higher energy state (e.g., T_2_), rather than T_1_ state. From here on, we term this subset of nonoptimal solutions as “state errors” for simplicity. As discussed below, convergence to states higher than T_1_ is sensitive to the reaction class, the initial SCF guess and the convergence algorithm used, and that these issues were not universally identified and corrected through wave function stability analysis. Failure to address such errors results in flawed benchmark results.

During our initial analysis of functional performance, we found several structures in the data set were prone to SCF convergence that resulted in spin densities corresponding to states lying higher in energy than the desired T_1_ excited state. These variationally incorrect electron densities led to large errors (>25 kcal/mol) across a subset of the reactions evaluated (**5, 6, 24, 25, 26, 36, 37**) for otherwise well-performing functionals. Such large errors result from comparing structures of one excited state to those of another excited state, e.g., the T_1_ state to the T_2_ state. State errors were only observed for structures in the triplet state, with both minima and transition state structures subject to erroneous electron density convergence. Ultimately, we were able to mitigate these errors by obtaining the variationally correct T_1_ energies for all species under study (see below). Nevertheless, the presence of multiple solutions to the SCF equations arose with such frequency that they are likely to be encountered by others when dealing with triplet species and should be similarly resolved to avoid sizable errors.

Across the data set, errors up to 26.4 (barrier heights) and 22.7 kcal/mol (thermochemistry) were observed relative to DLPNO–CCSD­(T)/CBS. These errors occurred when using both ORCA and Q-Chem software packages, and across nearly all functionals evaluated, except PW6B95. However, there is no reason to believe PW6B95 is immune, and a broader data set would likely reveal similar issues across all functionals.

Seven reactions (14% of the total data set) contained at least one erroneously converged calculation, particularly those featuring aryl- and vinyl-ketone moieties. A notable case is triplet acetophenone, which suffered from state errors in both minimum and transition state structures. This led to large thermodynamic errors, but barrier heights were artificially good data due to error cancellation (Figure 3A). Erroneous densities for minima corresponded to the T_2_ state (a π→π* excitation), whereas corrected densities corresponded to the lower energy T_1_ state (an n→π* transition).^93^ We hypothesize the prevalence of state errors in structures with these moieties may be due to the shared symmetry between the S_0_ and T_2_ states in aryl- and vinyl-ketones, as the initial guess is the same between restricted and unrestricted calculations and may be more similar to the S_0_, and thus the T_2_, rather than the T_1_ state. The primary qualitative difference between these two states is the spin densities: the T_1_ state of a simple ketone possesses equal spin in orthogonal orbitals on carbonyl carbon and oxygen atoms, whereas the T_2_ state of aryl- and vinyl-ketones, common functional groups in organic photosensitizers, has little appreciable spin density on the carbonyl carbon (Figure 3B). This visual difference in spin densities between the two states allows for easy diagnosis of such convergence issues. As such, we caution against “black box” approaches in computing excited states due to these potentially catastrophic errors. Since such errors cannot be predicted *a priori*, manual inspection of spin densities for triplet-state calculations can be helpful to ensure the lowest triplet state has been converged with KS-DFT.

**Figure 3:**
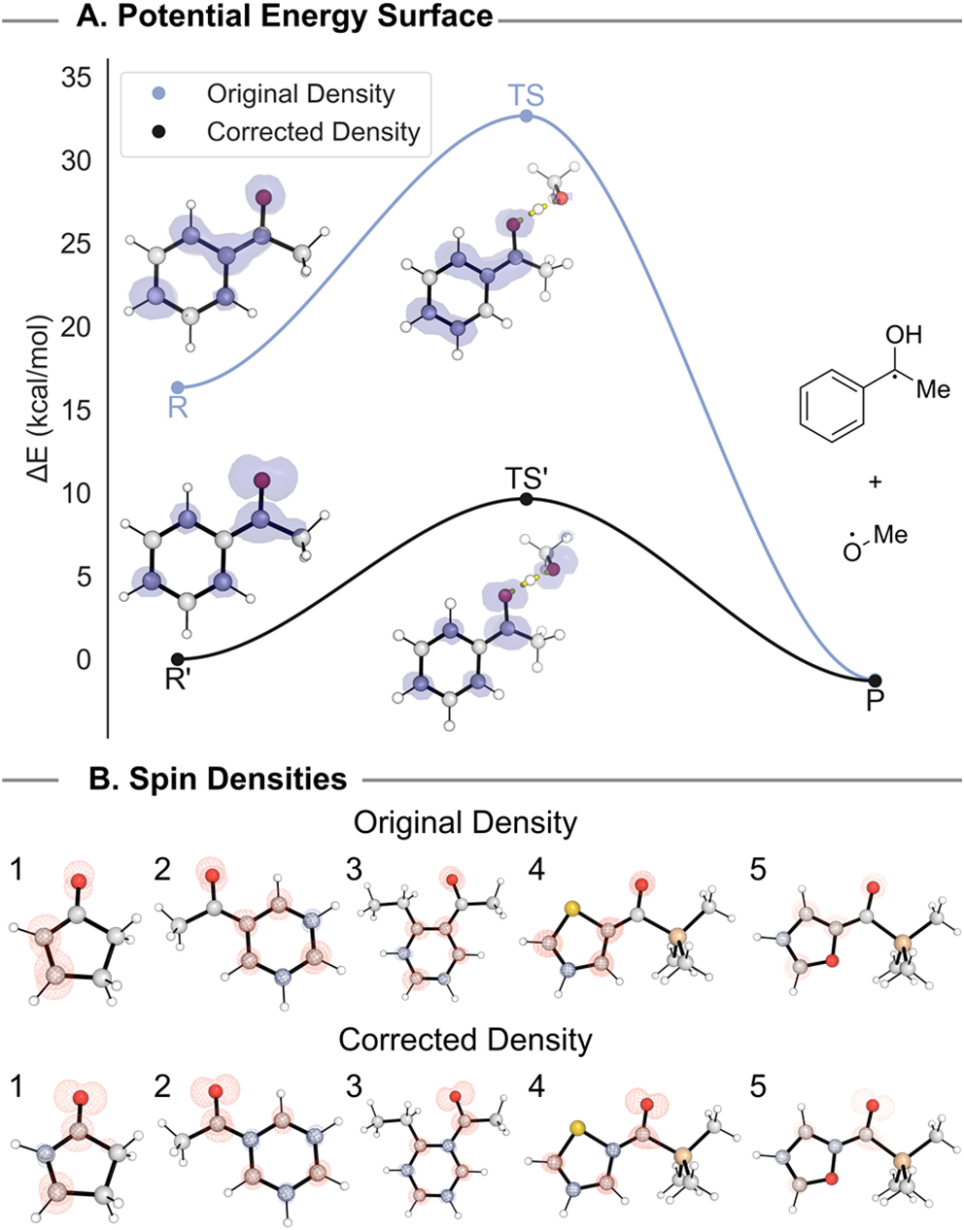
(A) Potential Energy Surface of a reaction with energies from erroneous (blue) and corrected (black) SCF convergence at the correct T_1_ geometry. Original single points were conducted using the ωB97M-V functional, with the initial guess read from the converged M06 density. (B) Spin densities of erroneous (top) and corrected (bottom) minima.

When analyzing the effect of these state errors, we realized the errors in kinetics and thermodynamics caused by incorrect SCF convergence were generally larger in magnitude than even the largest errors observed when comparing corrected densities against our reference values. Additionally, we observed no trends in which functionals most often resulted in state errors. Taking reaction 37 as an example, the effects of state errors are dramatic (Figure 4). Pure functionals seemingly outperform double hybrid and range-separated hybrid functionals (top panel), with barrier height errors ranging from 2.2–5.4 kcal/mol (for M06L-D4, B97M-V, TPSS-D4, BLYP-D4, PW91-D4, PBE-D4, and OLYP-D3­(BJ) functionals). However, with the corrected T_1_ densities (lower panel), the results are more closely aligned with the hierarchy of functionals expressed by Jacob’s Ladder, with hybrid, range-separated hybrid, and double hybrid functionals typically, but not always, outperforming pure functionals. Additionally, from Figure S5, it is easy to see a much tighter spread of data about the mean following correcting these errors in the data. Because of this, to evaluate functional performance and make recommendations for modeling reactions in the triplet state, incorrect densities have to be fixed.

**Figure 4:**
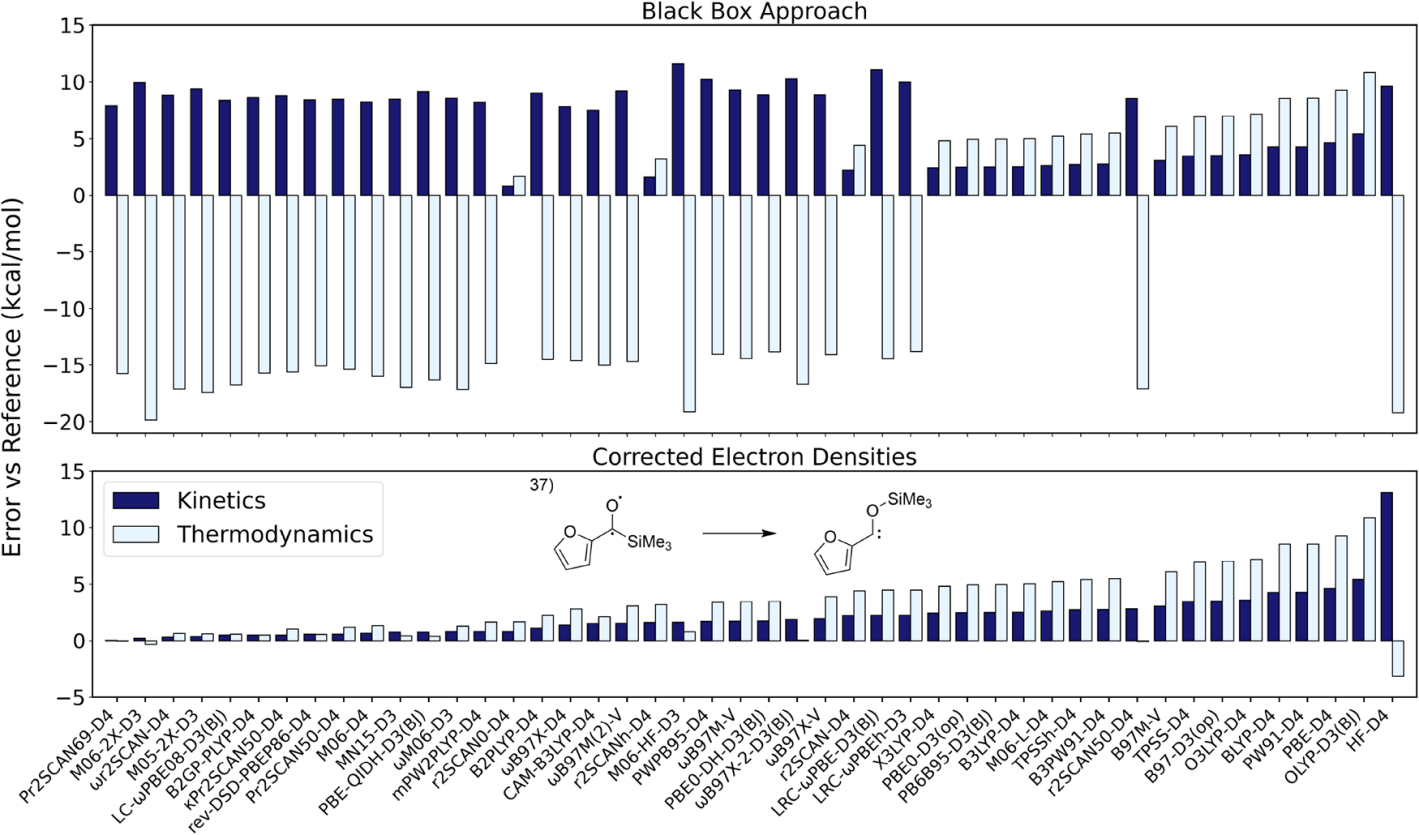
Comparison of energetic errors for reaction 37 when a black box approach is applied (top) and when state errors in SCF convergence are considered and corrected (bottom). Values in kcal/mol.

Initially, SCF stability analysis (i.e., evaluation of the eigenvalues of the electronic Hessian via matrix diagonalization in a manor akin to that used for TD-DFT calculations) was performed to rectify state errors. However, this approach proved to be inconsistent, as densities corresponding to higher energy triplet states still passed as stable wave functions. Following this, we explored the effect of the initial SCF guess, subjecting each species with an erroneous density to single point energy calculations using a subset of the functionals tested while varying the initial SCF guess (Table 1). This had a substantial impact, and we were able to converge as many as three distinct densities for each structure. We also found that, for this subset of the data, the default guesses for both ORCA (PModel) and Q-Chem (SAD) were some of the most likely to converge to a suboptimal density. Each yielded a state error for 11 of the 15 single point energy calculations, though PModel calculations never converged to the highest energy density observed for other initial guesses corresponding to the tentatively assigned T_3_ state. The AutoSAD guess for Q-Chem gave similar results, with nine erroneously converged densities. Additionally, all guesses in Q-Chem aside from AutoSAD yielded at least one T_3_ density. In ORCA, the HCore guess also yielded state errors for some structures, though at a lower rate than the default PModel guess. Finally, the Hueckel and PAtom guesses in ORCA yielded no state errors for this subset of the structures, though this trend may not continue should a larger data set be tested. Given the limited size of this data set and the magnitude of the issue at hand, a more thorough look at this issue and potential solutions is warranted.

**Table 1:**
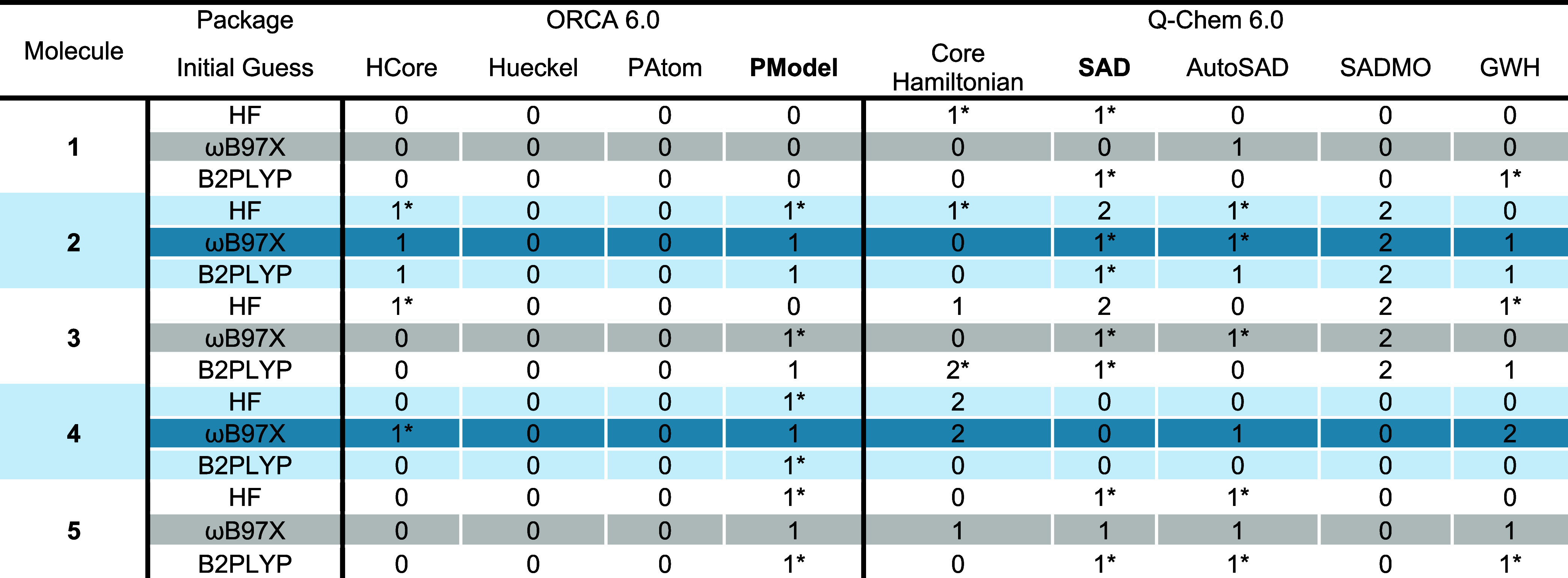
Results of Altering the Initial Guess for Molecules Subject to State Errors

* Wavefunction incorrectly found to be stable via SCF stability analysis. Values of 0, 1, and 2 correspond to SCF optimizations to the T_1_, T_2_, and T_3_ states, respectively. Default initial guess procedures in each software package are bolded.

After confirming alterations to the initial SCF guess could yield various electronic densities for the same structure, we set out to correct the state errors present in our data set. To generate the optimal initial guess for each system, converged SCF densities from functionals for which the correct T_1_ state was obtained (based on energy and visual inspection of spin density) were used as initial guesses for all other functionals for a given reaction, and the SCF convergence was allowed to proceed as usual. To our delight, this method led to the correct electron density convergence in all examples evaluated, and a more realistic, smaller range of errors for the affected reactions.

### Benchmarking Results

Following the calculation and state error correction of the TRIP50 data set, we began our evaluation of our selected set of density functionals. We first evaluated the performance of each functional across the entire data set of reactions (Figure 5). In this global analysis, two major trends stand out. First, our results are broadly aligned with other (ground state) benchmarks of reaction thermochemistry in that, as one proceeds up each rung of Jacob’s Ladder, the accuracy of a functional increases for functional forms at higher rungs.^24^ However, this is not universally accurate, and many hybrid and range-separated hybrid functionals perform comparably well to the best double hybrid functionals, including M05–2X-D3, M06–2X-D3, ωM06-D3, and the ωB97 family of functionals. Additionally, the worst performing double hybrid functional, PBE0-DH-D3­(BJ), was 22nd overall in its kinetic performance, putting it close to the median overall. Moreover, B3LYP-D4, one of the most commonly used “general purpose” hybrid functionals, performs comparably to many pure functionals, with an overall MAE of 3.9 kcal/mol in kinetics and 3.2 kcal/mol in thermodynamics. While the general trend of Jacob’s Ladder is observed, the results for each individual functional can vary substantially.

**Figure 5:**
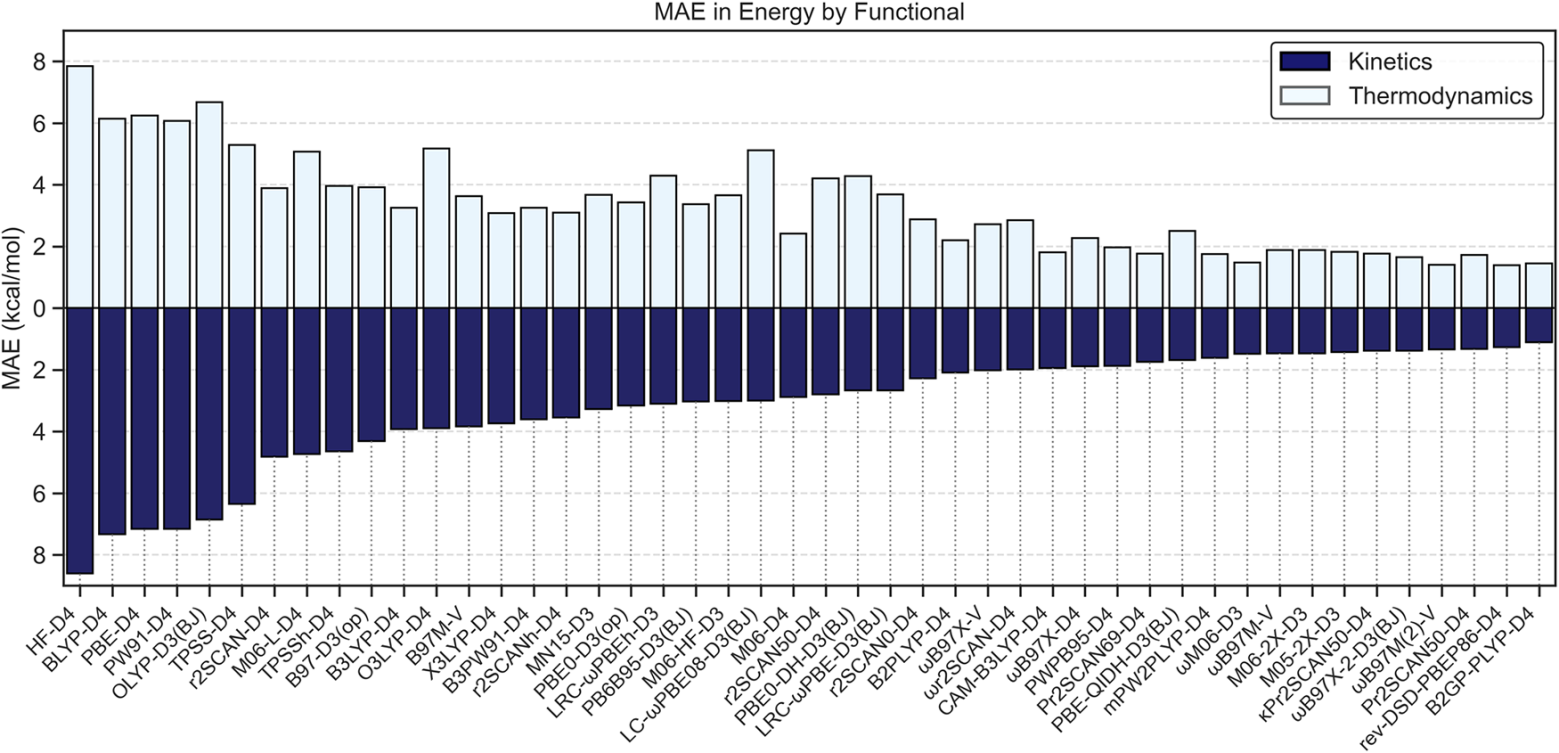
Bar chart of functional performance across the TRIP50 data set. Error in kinetics (dark blue) and thermodynamics (light blue) are reported as mean absolute error (MAE) in kcal/mol compared against reference values computed at DLPNO–CCSD­(T)/CBS.

The worst performing functionals belong to the GGA rung, followed by mGGA functionals. The average MAE across all GGA functionals was 7.1 kcal/mol in kinetics and 6.3 kcal/mol in thermodynamics, while those of mGGA functionals were 4.9 kcal/mol in kinetics and 4.5 kcal/mol in thermodynamics. These high errors are all prohibitive of any chemical explainability or predictability, severely limiting the use scope of such functionals. Given these large deviations in both kinetic and thermodynamic values relative to our reference values, we advise against the use of pure functionals in this domain.

Hybrid functionals overall performed much better. Functionals in the HGGA class presented average errors in kinetics/thermodynamics of 3.8/3.7 kcal/mol, respectively, while HmGGA functionals presented errors of 2.8/3.1 kcal/mol, respectively. Functionals in the RSH class performed slightly better on average than the HmGGA class, with errors in kinetics/thermodynamics of 2.2/2.9 kcal/mol, respectively. Finally, DH functionals saw a marked improvement over the performance of RSH functionals on average, with errors in kinetics/thermodynamics of 1.6/2.0 kcal/mol, respectively. We again stress, however, that while the average results of each class follow those expected by Jacob’s Ladder, individual functionals within each class, particularly those of the HmGGA, RSH, and DH classes, present outstanding results compared to the average results for the functional class.

Our second major observation from looking at performance across the data set was that most RSH functionals out-perform any nonrange-separated functionals from the same family. The RHS functional ωM06-D3 performs within 0.1 kcal/mol of the best non-DH functionals in kinetics while outperforming all non-DH functionals in thermodynamics, including a 0.3 kcal/mol improvement in thermodynamics MAE over the other best performing of Truhlar’s Minnesota functionals, M05–2X-D3. The third best performing of the B97-based functionals is ωB97M-V, only outperformed by its DH counterparts, ωB97X-2 and ωB97M(2).

The ωr2SCAN-D4 functional outperformed all of the non-DH SCAN functionals in both kinetics and thermodynamics. Of particular note, due to the prominence of B3LYP in literature studies, CAM-B3LYP-D4 outperforms its nonrange-separated counterpart by 2.0 kcal/mol in kinetics and 1.4 kcal/mol in thermodynamics on average across the TRIP50 data set, even outperforming DH B2PLYP-D4, adding further weight to arguments to supplant B3LYP as a “default” functional.

In the case of the PBE functionals, PBE0-D3­(op) outperforms in thermodynamics its RSH counterparts LRC-ωPBE08-D3­(BJ), LRC-ωPBEh-D3(0), and LRC-ωPBE-D3­(BJ) by 1.7, 0.9, and 0.3 kcal/mol, respectively, though each RSH slightly outperformed PBE0-D3­(op) in kinetics across the entire data set by 0.2, 0.1, and 0.5 kcal/mol, respectively. The overall results of RSH functionals generally outperforming any nonrange-separated functionals from the same family is consistent with other studies of open shell systems.^30,31^

Overall, the trends observed in these averaged results of performance across the TRIP50 data set are consistent with other benchmarks including thermodynamic and kinetic data.^24,25^ In particular, the errors of the top performing functionals in such studies are typically around the 2–3 kcal/mol range, as observed here. The top performing functionals for most of the classes were also consistent with other benchmarks of this nature, with Truhlar’s Minnesota functionals and the Head-Gordon ωB97 family of functionals giving cost-effective accuracy compared to Coupled-Cluster methods.^24^ Of note, given that many double hybrid functionals are fairly new, thermodynamic and kinetic benchmarks are relatively sparse. As such, our results of the performance of these functionals in comparison to other functionals across all classes are some of the first to be reported. Additionally, the ωM06-D3 functional, while performing remarkably in this study for both kinetics and thermodynamics of triplet transformations, remains underutilized in the broader literature, and in fact has been hitherto unseen in a benchmark of this nature. We point out that these functionals, the top performing DH functionals and ωM06-D3, substantially outperform other functionals widely used to study organic transformations. In particular, B3LYP, PBE0, and TPSSh hybrid functionals are widely used, and are all less accurate on our data set than ωM06-D3 by ≥ 1.7 kcal/mol for both kinetics and thermodynamics.

Following our analysis of each functional’s performance on the entire data set, we sought to further analyze the data spread and the performance of the top functional in each category: B2GP-PLYP-D4, ωB97M-V, M05–2X-D3, PBE0-D3­(op), B97M-V, and OLYP-D3 (Figure 6A). To make more meaningful conclusions based on forward thermochemical data alone, the direction of forward reactivity was defined as the direction for which the reference thermodynamics were exothermic. As such, reactions 1, 10, 11, 20, 27, 38, 39, 40, and 41 had their reactants and products exchanged for this analysis. Not only do the functionals higher on Jacob’s ladder have lower MAEs, but they also present close alignment with reference values across the entire range of values computed for both kinetics and thermodynamics, resulting in a high *R* ^2^ for these functionals. Following the trend of the MAE data, the best performing functionals in the HmGGA, RSH, and DH classes all perform comparably across the data set. In terms of kinetics, the DH functional B2GP-PLYP-D4 performed the best, with an *R* ^2^ of 0.945 across the entire data set when plotted vs the reference values for each reaction. The RSH functional ωB97M-V and the HmGGA functional M05–2X-D3 followed closely behind, with *R* ^2^ in kinetics of 0.906 and 0.881, respectively. The thermodynamic results for these three functionals agree well with the reference values, and the functionals all performed comparably on these data. Thermodynamics *R* ^2^ values for these DH, RSH, and HmGGA were 0.967, 0.967, and 0.962, respectively. This close alignment with the reference data across the entire data set for both thermodynamics and kinetics, combined with the low MAE for each of these three functionals for both values, further cements our recommendation for the use of these functionals when modeling organic triplet reactions.

**Figure 6:**
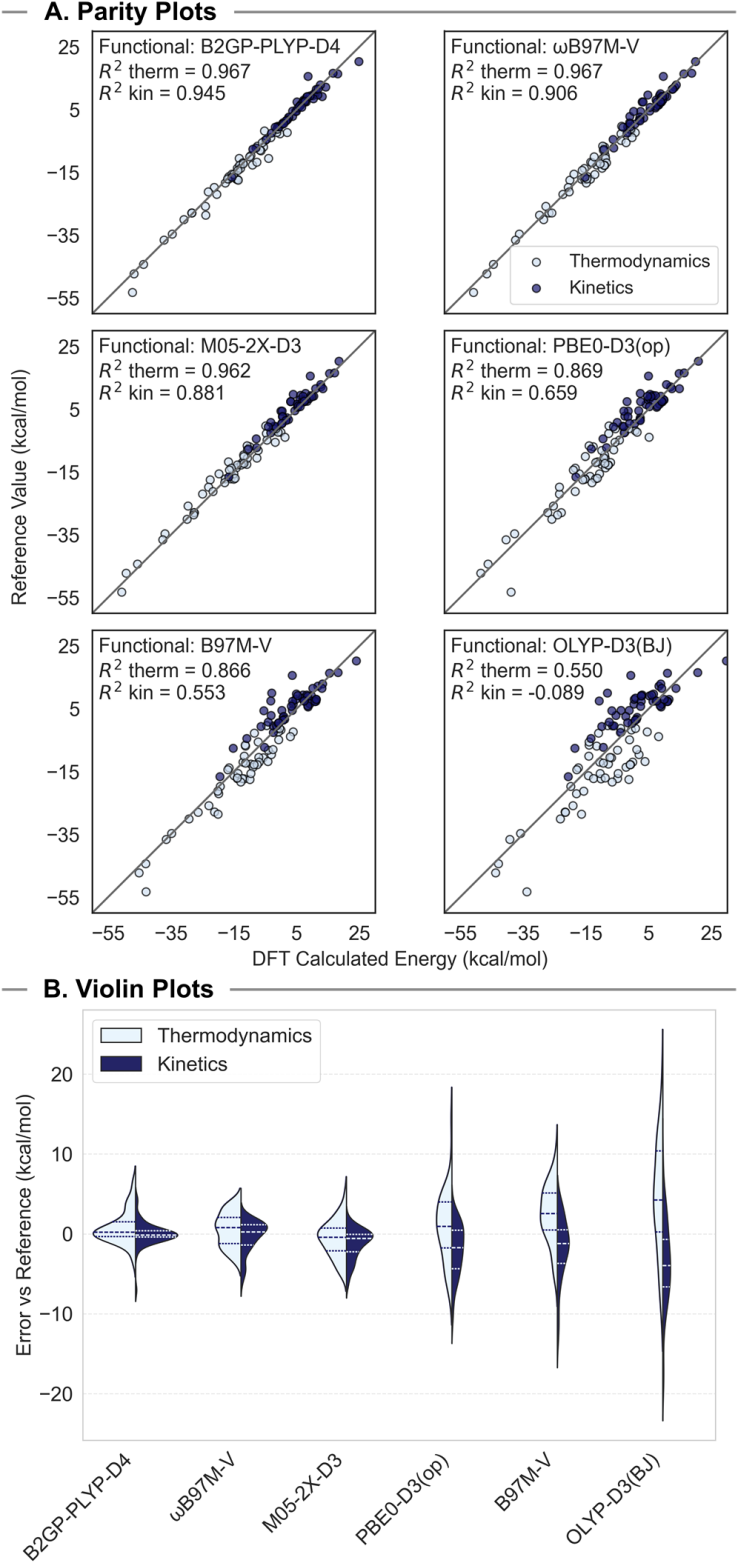
(A) Scatter plots of kinetic (dark blue) and thermodynamic (light blue) energies obtained for the top functional in each functional class vs calculated reference values computed at DLPNO–CCSD­(T)/CBS. (B) Violin plots of performance for the top functionals in each functional class. The width of each plot at a given *Y* value corresponds to the density of data at that value. Dashed lines on each plot designate the mean and interquartile range (IQR).

Performance began to drop when looking at the best HGGA, mGGA, and GGA functionals PBE0-D3­(op), B96M-V, and OLYP-D3, respectively. In terms of thermodynamics, PBE0-D3­(op) and B97M-V perform reasonably, with *R* ^2^ values of 0.869 and 0.866, respectively, when plotted against the reference values for these reactions. However, there is a sharp decline in performance for the GGA functional OLYP-D3, with an *R* ^2^ in thermodynamics of 0.550. Additionally, these functionals’ performance drops substantially when compared to the top three functionals discussed earlier when the kinetics data are considered, as the best performing HGGA, mGGA, and GGA functionals reported *R* ^2^ values in kinetics of 0.659, 0.553, and −0.089, respectively. These *R* ^2^ values show moderate to no correlation between the barrier heights computed using these functionals when compared to the reference values.

Examining these results further using violin plots, the similarity in performance between the top functionals in the DH, RSH, and HmGGA classes can be readily observed (Figure 6B). Each has a tightly packed distribution for both thermodynamics and kinetics, and each quartile for these functionals falls within ± 2.2 kcal/mol error. The most extreme errors lie within 7.0 kcal/mol for each of these three functionals. However, the data spread widens drastically when visualizing the results of the top HGGA, mGGA, and GGA functionals. For each of these functionals, the mean is no longer centered about zero, with a much larger interquartile range (IQR), with a range of 3.5 and 4.7 kcal/mol (barriers and thermochemistry) for PBE0-D3­(op), 5.1 and 4.2 kcal/mol for B97M-V, and 5.3 and 7.5 kcal/mol for OLYP-D3. Additionally, for both the top mGGA and GGA functionals the thermochemistry of >2/3 of the reactions is overestimated, such that zero error lies outside of the IQR. For OLYP-D3, the same is true for kinetics, though the barriers were underestimated instead of overestimated. The data points with the largest errors are also more pronounced for these functionals, with errors >10 kcal/mol for both thermodynamics and kinetics, and with OLYP-D3 reporting errors exceeding 18 kcal/mol for both kinetics and thermodynamics.

To further delve into the specific performance of each functional on different reaction classes, we visualized the performance of the top functionals in each class on each of the reaction classes in the TRIP50 data set (Figure 7). Certain transformations stand out as more difficult to describe using DFT methods. All functionals show the largest error in kinetics for triplet state HAT reactions, except in the case of B97M-V, for which it is the second largest. Additionally, C–Hal and C–O activation energy barriers seem to be more prone to errors than other reaction classes. In terms of thermodynamics, C–S and C–O reactions appear to be difficult to describe for all functionals when compared to the other reaction classes. However, results for the other reaction classes vary substantially by specific functional. In thermodynamics, B2GP-PLYP-D4 and M05–2X-D3 find their best performance in Si–X reactions, while all other top performing functionals give the highest or second highest errors for this class over other reaction classes. In particular, ωB97M-V displays its highest error overall for Si–X reaction thermodynamics at 3.6 kcal/mol, which is 1.5 kcal/mol higher than its second highest thermodynamic error. This highlights the importance of selecting functionals based on the specific chemistry being modeled, rather than the general type of chemistry, and potentially performing independent benchmarking on the system of interest.

**Figure 7:**
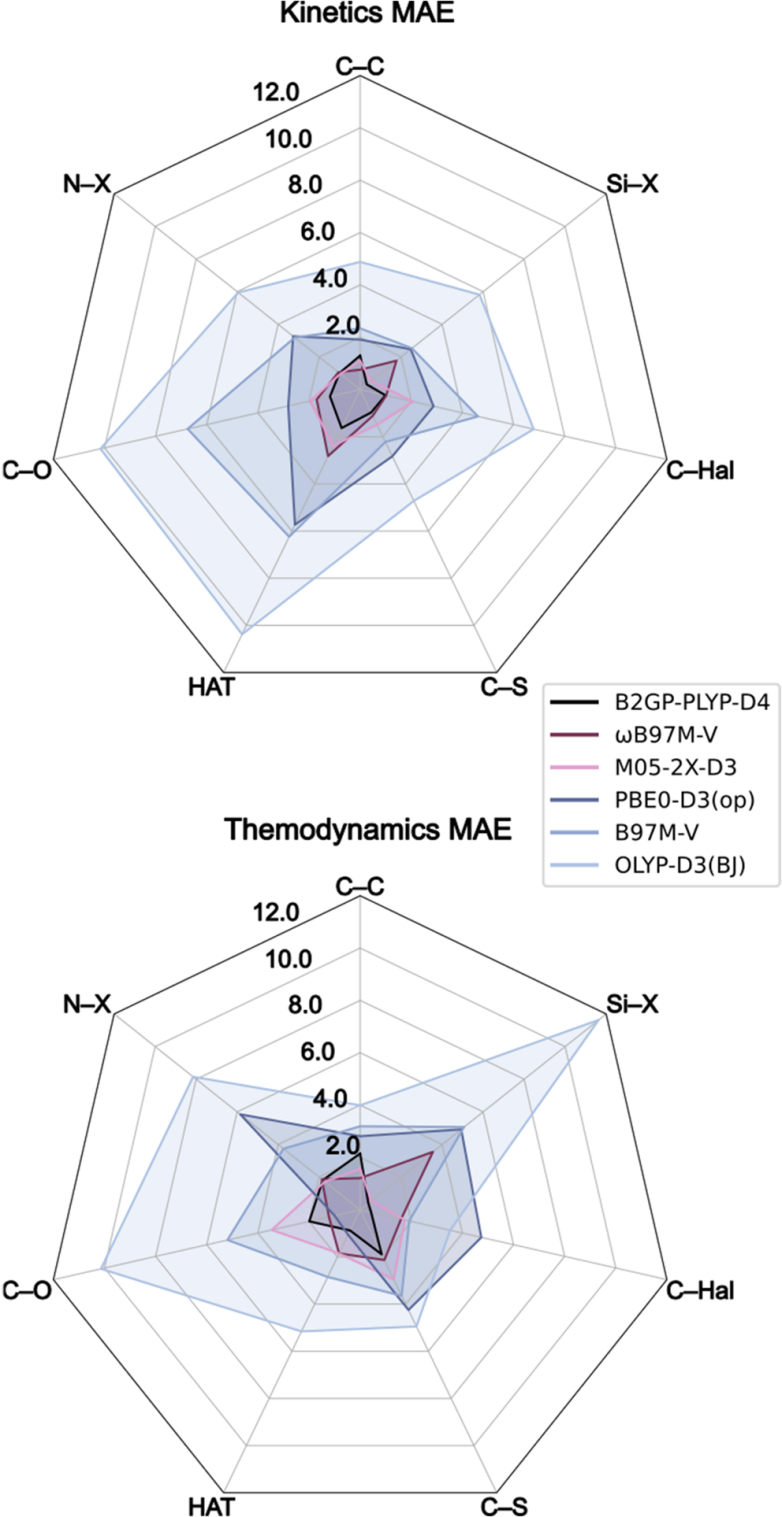
Radar plots of mean absolute error (MAE) for the top performing functional in each class in kinetics (top) and thermodynamics (bottom). The data are split into the seven reaction classes. Values in kcal/mol.

Taking a closer look at the B2GP-PLYP-D4, ωB97M-V, and M05–2X-D3 functionals (Figure 7), we see that each of these functionals perform within 1.1 kcal/mol of each other for most reaction classes in both thermodynamics and kinetics. However, in cases such as for HAT kinetics and thermodynamics and C–Hal thermodynamics, there is a substantial increase in performance when choosing to use the DH B2GP-PLYP-D4 over the other top performing functionals. In other cases, such as for N–X kinetics and thermodynamics, all three functionals perform within 0.1 kcal/mol of each other. Overall, the top performing DH, RSH, and HmGGA functionals generally perform comparably. Additionally, given that these three functionals perform well across all metrics tested and across all functional types, one should strongly consider these when modeling triplet reactivity, particularly if it is of a reaction class not represented by the classes of reactions tested in this study.

## Conclusion

Based on the data presented in this study, we make the following recommendations for modeling organic triplet reactions using DFT methods:

- Foremost, when modeling reactions in the triplet state, UKS-DFT cannot be treated as a black box. One must ensure the proper density/wave function is obtained following SCF convergence, either by manual inspection of the spin density or by comparing results using different initial guesses for the SCF convergence. Wave function stability analysis in the triplet state is also advisible, although this also cannot be used in black box fashion to identify all stability issues. Without such precautions, catastrophic errors in barrier height and thermochemistry >20 kcal/mol can arise. The issues particularly affect, although are not limited to, transformations involving conjugated carbonyl groups.
- Across all reactions, general improvement when ascending Jacob’s Ladder was observed, particularly when the performance of functionals from each rung are averaged (see SI for details). This suggests that, should it be computationally feasible, the use of a functional from a higher rung typically yields more accurate results. However, many individual functionals do not follow this trend and perform extraordinarily well or poorly compared to functionals of the same class. In particular, ωM06, M06–2X, M05–2X, and the ωB97 family of functionals all perform well while the popular functionals B3LYP and TPSSh perform poorly relative to other functionals in our tests.
- In general, results of MAE in thermodynamics trend with those for kinetics. This suggests that functionals generally perform equally well for both sets of values, and that choosing a functional that provides good thermodynamic values will generally also yield good kinetic values.
- Certain reaction classes, such as HAT and reactions involving C–O bond forming or breaking were found to give worse results and may require more advanced functionals to obtain accurate results. In such cases, we recommend the use of a DH functional, if computationally feasible.
- Functionals of the GGA, mGGA, and HGGA classes exhibited extreme errors and little correlation with DLPNO–CCSD­(T)/CBS results. Such deviations could exceed 20 kcal/mol, with correlation coefficients as low as 0.1 against reference values for even the best functionals in these classes.

Given these results, we discourage the use of functionals in the GGA, mGGA, or HGGA classes due to their large errors and low correlation with reference values observed in this study, particularly if accurate kinetic values are required. Additionally, the popular functionals B3LYP, PBE0, and TPSSh are outperformed by other functionals with similar performance costs, and we similarly discourage the continued reliance on these functionals due to their inability to accurately describe reactions of this type. In particular, CAM-B3LYP substantially outperforms B3LYP for both kinetics and thermodynamics and thus presents itself as a good alternative over the former.

As such, for the best overall performance and robustness, we recommend the use of the DH functionals B2GP-PLYP revDSD-PBEP86, Pr2SCAN50, ωB97M(2), ωB97X-2, κPr2SCAN50. However, given the higher cost associated with the use of DH functionals, along with the marginal-at-best improvement in performance compared to RSH and HmGGA functionals, the use of functionals of the latter two classes is encouraged as they strike a good balance accuracy and computational cost throughout this study. As such, the use of the range separated HmGGA ωM06 and ωB97M functionals, as well as the M06–2X and M05–2X, is recommended here.

For the identification and alleviation of state errors, we recommend the use of internal stability analysis when performing any UKS calculation. As a sanity check, particularly when computed energetic values vary wildly from expected results, we recommend changing the initial SCF guess to Hueckel or PAtom, if one is using ORCA, or to SADMO, if one is using Q-Chem, as these guesses appear to better align with the triplet wave function and thus yield lower rates of state errors. Finally, SCF metadynamics is a technique developed for identifying global minimum SCF solutions and thus is useful for identifying when a state error is present and for obtaining the correct SCF solution for such cases. We recommend solving for as many as 10 solutions using this technique, as we have found this most consistently alleviates issues in resolving the lowest energy electronic state.

The present study is limited to a data set of 50 elementary reactions. The elemental coverage excludes certain heteroatoms (e.g., B or P atoms) and is limited to the 7 reaction types shown in Figure 2. Additionally, we focused here solely on organic species, omitting reactions involving metals or transition metals. Finally, due to computational cost associated with DLPNO–CCSD­(T)/CBS reference calculations, the data set was limited to systems including 20 or fewer heavy atoms. However, all DFT functionals tested herein are readily applicable to systems much larger than those tested. With these limitations in mind, we believe, for computational studies of organic triplet reactions, the conclusions made are robust and supported by our data presented herein.

## Supplementary Material









## Notes

TRIP50 data set (CSV)
TRIP50 original densities (CSV)
XYZ coordinates (ZIP)
Further technical details, tabulated results before and after correction of electron densities (PDF)
