# Supplementary material for: Fundamental Study of Density Functional Theory Applied to Triplet State Reactivity: Introduction of the TRIP50 Data Set
Source: J Chem Theory Comput. 2026 Mar 19;22(7):3530–42. doi: 10.1021/acs.jctc.6c00144 (PMC13085248; doi:10.1021/acs.jctc.6c00144)
Supplement: Supplementary file 4 [file ct6c00144_si_004.pdf]

Supporting Information for

Fundamental Study of Density Functional Theory Applied to Triplet State Reactivity: Introduction of the TRIP50 Dataset

William B. Hughes[a], Mihai V. Popescu[a],* Robert S. Paton[a]*

[a]Department of Chemistry, Colorado State University, Ft. Collins, CO, 80523-1872, United States

* Emails: mihai-viorel.popescu@univie.ac.at, rpaton@colostate.edu

Contents

1. Details of Selected Density Functionals S3
2. Calculation of Reported Errors S3
3. Ensuring Reliability in the Reference Calculations S4
3a. Conformational Sampling S4
3b. Evaluation of Reference Calculations for State Errors S5
3c. Testing for Multireference Character S5
4. Evaluation of Q-Chem Methods for Avoiding State Errors S6
5. Additional Benchmark Results S8
6. References S15

1. Details of Selected Density Functionals

Table S1: Details on density functionals investigated, software in which calculations using that functional were carried out, dispersion correction applied, and MAE for barrier height and thermochemistry.

Theory	Type	Software	Dispersion	MAE Kinetics (kcal/mol)	MAE Thermo (kcal/mol)	Reference
ωB97X-2-D3(BJ)	DH	ORCA	D3BJ	1.381	1.645	1
Pr2SCAN50-D4	DH	ORCA	D4	1.325	1.726	2
Pr2SCAN69-D4	DH	ORCA	D4	1.752	1.76	2
PWPB95-D4	DH	ORCA	D4	1.881	1.959	3
rev-DSD-PBEP86-D4	DH	ORCA	D4	1.263	1.382	4
B2GP-PLYP-D4	DH	ORCA	D4	1.105	1.441	5
B2PLYP-D4	DH	ORCA	D4	2.092	2.196	6
κPr2SCAN50-D4	DH	ORCA	D4	1.383	1.766	2
mPW2PLYP-D4	DH	ORCA	D4	1.619	1.755	7
PBE0-DH-D3(BJ)	DH	ORCA	D3BJ	2.671	4.273	8
PBE-QIDH-D3(BJ)	DH	ORCA	D3BJ	1.686	2.498	9
ωB97M(2)-V	DH	Q-Chem	VV10	1.343	1.407	10
CAM-B3LYP-D4	RSH	Q-Chem	D4	1.945	1.814	11
LC-ωPBE08-D3(BJ)	RSH	Q-Chem	D3BJ	3.007	5.118	12
LRC-ωPBE-D3(BJ)	RSH	Q-Chem	D3BJ	2.667	3.689	13
LRC-ωPBEh-D3	RSH	Q-Chem	D3	3.109	4.287	14
ωB97M-V	RSH	Q-Chem	VV10	1.47	1.879	15
ωB97X-D4	RSH	Q-Chem	D4	1.887	2.275	16
ωB97X-V	RSH	Q-Chem	VV10	2.017	2.717	16
ωM06-D3	RSH	Q-Chem	D3	1.486	1.47	17
ωr2SCAN-D4	RSH	ORCA	D4	1.997	2.841	2
M05-2X-D3	HmGGA	Q-Chem	D3	1.422	1.818	18
M06-D4	HmGGA	Q-Chem	D4	2.882	2.411	19
M06-2X-D3	HmGGA	Q-Chem	D3	1.468	1.878	19
M06-HF-D3	HmGGA	Q-Chem	D3	3.014	3.657	20
MN15-D3	HmGGA	Q-Chem	D3	3.274	3.672	21
PB6B95-D3(BJ)	HmGGA	Q-Chem	D3BJ	3.035	3.372	22
r2SCAN0-D4	HmGGA	ORCA	D4	2.285	2.875	23
r2SCAN50-D4	HmGGA	ORCA	D4	2.807	4.2	23
r2SCANh-D4	HmGGA	ORCA	D4	3.545	3.086	23
TPSSh-D4	HmGGA	Q-Chem	D4	4.647	3.961	24
B3LYP-D4	HGGA	Q-Chem	D4	3.92	3.248	25,26
B3PW91-D4	HGGA	Q-Chem	D4	3.602	3.256	25
B97-D3(op)	HGGA	Q-Chem	D3OP	4.314	3.914	27
O3LYP-D4	HGGA	Q-Chem	D4	3.9	5.172	28
PBE0-D3(op)	HGGA	Q-Chem	D3OP	3.16	3.427	29
X3LYP-D4	HGGA	Q-Chem	D4	3.741	3.076	30
B97M-V	mGGA	Q-Chem	VV10	3.836	3.627	31
M06-L-D4	mGGA	Q-Chem	D4	4.737	5.07	32
r2SCAN-D4	mGGA	ORCA	D4	4.818	3.892	33
TPSS-D4	mGGA	Q-Chem	D4	6.348	5.281	34
BLYP-D4	GGA	Q-Chem	D4	7.337	6.135	35–37
OLYP-D3(BJ)	GGA	Q-Chem	D3BJ	6.863	6.674	36–38
PBE-D4	GGA	Q-Chem	D4	7.169	6.241	39
PW91-D4	GGA	Q-Chem	D4	7.158	6.063	40
HF	Wavefunction	ORCA	D4	8.602	7.851	41,42

2. Calculation of Reported Errors

For errors averaged across multiple reactions, values are reported as mean absolute error (MAE), calculated as

$$MAE = \frac{1}{n}\sum_{i=1}^{n}\left|\Delta E_{Calculated} - \Delta E_{Reference}\right| \quad (1)$$

where n, in this case, is the number of reactions over which the average is being taken, $\Delta E_{Calculated}$ is the thermochemical value, either for barrier height or reaction energy, calculated using a given functional for one reaction, and $\Delta E_{Reference}$ is the reference value for that reaction calculated using DLPNO-CCSD(T)/CBS(3,4).

For kinetic values reported, both forward and reverse barrier heights were calculated

$$\Delta E_{FBH} = \sum E_{Reactants} - E_{TS} \quad (2)$$

$$\Delta E_{RBH} = \sum E_{Products} - E_{TS} \quad (3)$$

To not weight one sense of reaction over the other, errors in kinetics were calculated as the average of the absolute errors in both forward and reverse barrier heights, as

$$Error = \frac{\left|\Delta E_{FBH_{Calculated}} - \Delta E_{FBH_{Reference}}\right| + \left|\Delta E_{RBH_{Calculated}} - \Delta E_{RBH_{Reference}}\right|}{2} \quad (4)$$

Thus, unless specified, error values in kinetics reported in the manuscript are always positive.

3. Ensuring Reliability in the Reference Calculations

3a. Conformational Sampling

Conformational sampling was performed for all structures using a combination of RDKit[43] generated geometries and manual conformation sampling. All generated conformers were optimized in the Gaussian 16[44] software package using the ωB97X-D[45]/def2-TZVP[46] level of theory, and the lowest energy conformer of each structure was selected for further analysis. To examine the efficacy of using RDKit generated conformers as a starting point for DFT optimization in the triplet state, conformational sampling using the GOAT algorithm[47] and the GFN2-XTB semiempirical method[48] in the ORCA 6.0.0[49] software package was performed (as suggested by the reviewer). We selected one of the most conformationally labile structures in our dataset, 48-P1. The GOAT algorithm found 310 conformers, roughly double that of RDKit, which found 157. After subjecting these initial conformers to geometry optimization (ωB97X-D/def2-TZVP level of theory), 148 unique conformers were found using the geometries from GOAT, while using the initial geometries generated by RDKit we obtained 135 unique conformers. Most importantly, the lowest energy conformers (following DFT optimization) generated from each workflow are essentially identical (Figure S1). Given the majority of structures in the TRIP50 dataset are conformationally rigid and possess as few as 1 or 2 conformers, the same conformer for each structure was used across all calculations, and RDKit sampling was further augmented with manual conformational sampling, we believe this sampling method was sufficient for the benchmarking task at hand.

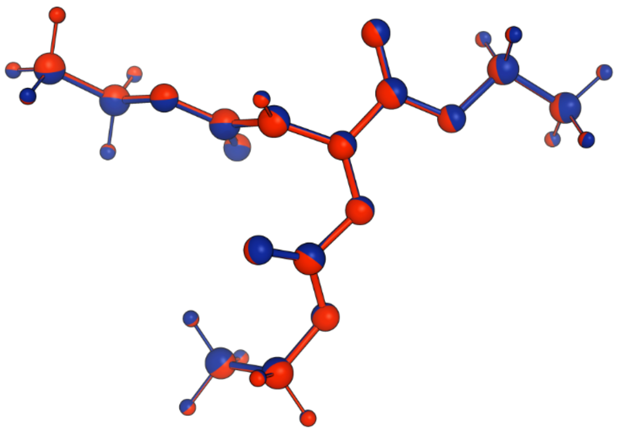

Figure S1: Final lowest energy geometries generated from workflows employing the GOAT (blue) and RDKit (red) conformational sampling methods.

3b. Evaluation of Reference Calculations for State Errors

Calculations using the DLPNO-CCSD(T) reference method use the HF method for generating the initial reference wavefunction. Given HF calculations are subject to state errors, we set out to ensure the reference wavefunctions used for each DLPNO-CCSD(T) calculation were void of state errors. To do this, HF single point calculations were performed for all structures in the TRIP50 dataset using each of the 4 initial guesses tested in ORCA. For structures in which multiple solutions were found for the HF wavefunction, the lowest energy wavefunction was manually compared against that of the reference level of theory to determine if the reference calculation indeed found the lowest energy solution. In all but one case, the lowest energy HF wavefunction matched that found in the DLPNO-CCSD(T) calculation. For the calculations of 7-R1, the HF wavefunction converged using the PAtom initial guess was found to be lower in energy than that used as a reference for the DLPNO-CCSD(T) calculation. However, after reading in this HF solution as an initial guess for a DLPNO-CCSD(T) calculation, it was discovered that there was a reordering of states between the HF and DLPNO-CCSD(T) methods, and that the original converged wavefunction was still lower in energy on the higher-fidelity surface. In summary, no state errors were found in the reference calculations.

3c. Testing for Multireference Character

To ensure the efficacy of the Coupled Cluster reference level of theory, a single-reference wavefunction method, for the chosen reactions, testing was carried out on each of these reference structures to determine the level of multireference character (Table S2). The %TAE(T) and T_1 diagnostic values were computed at the DLPNO-CCSD(T)/def2-TZVPPD and DLPNO-CCSD(T)/CBS(3,4) level of theories, respectively. Structures with a %TAE(T) values of >2.5% and a T_1 diagnostic value of > 0.02 were flagged as being potentially multireference. Wavefunctions for flagged structures were then computed using the complete active space self-consistent field (CASSCF) method in the ORCA 6.0.0 software package with the def2-SVP basis set. Structures for which the highest contributing configuration comprised lower than 90% of the total wavefunction were excluded from the TRIP50 dataset as a precaution.

In total, eight structures were flagged as potentially multireference. Following the CASSCF calculations, structures 8-R2, 11-P2, 27-R2, 27-TS, 28-TS, and 42-R1 were found to possess little to no multireference character. For the majority of these structures, >97% of the CASSCF wavefunction was made up of a single configuration. However, two structures were found to have potential multireference character, deemed 51-R1 and 51-TS, with the primary configuration contributing 84% and 83% to the CASSCF wavefunction, respectively. The reaction containing these structures, deemed 51, was thus removed from the dataset as a precaution (Figure S2).

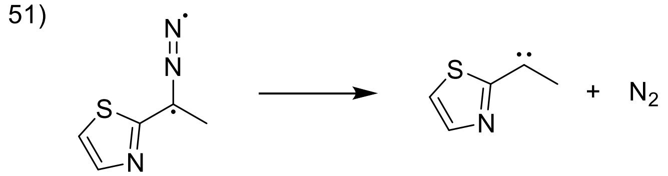

Figure S2: Reaction 51, an extrusion of N_2 to form a triplet carbene.

Table S2: Results of multireference tests for potentially multireference structures.

Name	TAE[CCSD] (Hartree)	TAE[CCSD(T)] (Hartree)	%TAE[(T)]	T1 diagnostic	CASSCF Electrons	CASSCF Orbitals	Highest Contribution
51-TS	1332.640238	1369.893904	2.72	0.024	12	12	0.83
51-R1	1344.114567	1380.384566	2.63	0.025	10	10	0.84
11-P2	473.8905561	492.7333888	3.82	0.024	7	6	0.92
42-R1	1055.731987	1085.904783	2.78	0.021	10	8	0.94
28-TS	545.4708303	560.01506	2.60	0.030	6	6	0.97
27-TS	354.3428922	369.2694789	4.04	0.047	6	6	0.98
27-R2	141.0524582	154.3242698	8.60	0.023	4	4	0.98
8-R2	104.6281536	108.8532257	3.88	0.021	10	6	1.00

4. Evaluation of Q-Chem Methods for Avoiding State Errors

To determine a workflow for discovering and avoiding state errors, optimization algorithms and initial SCF guesses available in the Q-Chem 6.0.2 software package were combinatorially evaluated for their rate of state error convergence on the set of 5 minima structures evaluated in Table 1. A total of 750 single point calculations were performed across the 5 structures, 3 methods (HF, ωB97X, and B2PLYP), 5 initial SCF guesses, and 9 optimization algorithms with the addition of the SCF metadynamics[50] technique for locating multiple minima on the SCF surface (Table S3). Calculations employing the SCF metadynamics technique used the default initial SCF guess, SAD, and convergence algorithm, DIIS, available in Q-Chem. The calculations were set to converge 10 distinct SCF minima, though many failed to converge the later minima. Any calculation that found the lowest energy wavefunction prior to failing to converge or converging 10 minima were considered to have avoided a state error, while those that only converged higher energy wavefunctions were considered to have converged a state error. Across all SCF metadynamics calculations, no minima were located that possessed a lower energy to those used in the TRIP50 benchmarking dataset for that computational method.

Across all methods tested, the rate of state errors in the converged wavefunctions was 34.6%. The rate for each DFT method were approximately equal, with 30.6% of ωB97X and 30.3% of

B2PLYP calculations yielding state errors. The wavefunction method HF had higher rates of state errors, with 42.6% of calculations using this method yielding such errors. The SADMO initial SCF guess provided the lowest rate of state errors, with only 13.1% of calculations using this initial guess yielding such errors. The Core Hamiltonian, GWH, and AUTOSAD guesses had rates of errors of 26.1%, 33.6%, and 42.5%, respectively, while the default guess, SAD, had the highest rate of state errors, with 56.9% of calculations using this initial guess yielding such errors.

When evaluating the effect of optimization method on the rate of state errors, SCF metadynamics stands out as being far and away the least error-prone method, with only 3 cases of state errors present across the 75 calculations using this method, a 4.0% error rate. This result is to be expected, as the method was developed for locating multiple minima on the SCF surface, and thus it can be readily used for cases in which the first SCF solution found may not be the global minimum. Following SCF metadynamics, the GDM_LS and GMD algorithms had state error rates of 29.3% and 29.4%, respectively, and the RCA and ADIIS algorithms had the next lowest error rates at 33.3% each. The highest error rates were found for the L_BGFS algorithm, at 49.3%, and the default algorithm DIIS, which had an error rate of 54.7%. Finally, conclusions regarding the DM, Newton, and Roothaan convergence algorithms cannot be made, as a high number of calculations (≥40%) using these algorithms failed to converge.

Table S3: Results of altering the initial guess and optimization method for molecules subject to state errors in the Q-Chem 6.0.2 software package. The default guess initial guess and optimization method are bolded. Values of 0 represent convergence to the T1 state, values of 1 represent convergence to a higher state (i.e. a state error), and values of X represent failure to converge the SCF.

Molecule		**1**			**2**			**3**			**4**			**5**		
Optimization Method	Initial Guess	HF	ωB97X	B2PLYP	HF	ωB97X	B2PLYP	HF	ωB97X	B2PLYP	HF	ωB97X	B2PLYP	HF	ωB97X	B2PLYP
**DIIS**	Core Hamiltonian	1	0	0	1	0	0	1	0	1	1	1	0	0	1	0
	**SAD**	1	0	1	1	1	1	1	1	1	0	0	0	1	1	1
	AUTOSAD	0	1	0	1	1	1	0	1	0	0	1	0	1	1	1
	SADMO	0	0	0	1	1	1	1	1	1	0	0	0	0	0	0
	GWH	0	0	1	0	1	1	1	0	1	0	1	0	0	1	1
ADIIS	Core Hamiltonian	0	0	1	0	0	0	0	0	1	0	0	0	0	0	0
	**SAD**	1	0	1	1	1	1	1	1	1	0	0	0	1	1	1
	AUTOSAD	0	1	0	1	1	0	0	1	0	0	1	0	1	1	1
	SADMO	0	0	0	0	0	0	0	0	0	0	0	0	0	0	0
	GWH	1	1	0	0	0	0	1	0	0	0	0	0	0	1	0
DM	Core Hamiltonian	X	X	X	X	X	X	0	0	0	0	X	X	0	1	X
	**SAD**	1	0	1	1	0	0	1	0	1	0	0	0	1	0	0
	AUTOSAD	X	X	X	X	X	X	X	X	X	X	X	X	X	X	X
	SADMO	0	0	1	1	0	1	1	0	1	0	1	0	0	0	1
	GWH	1	X	X	X	X	X	1	1	0	0	0	X	0	0	0
GDM_LS	Core Hamiltonian	1	0	0	1	0	0	0	1	0	1	0	0	1	0	0
	**SAD**	1	0	1	1	0	0	1	1	1	0	0	0	1	0	1
	AUTOSAD	0	1	0	1	0	0	0	1	0	0	0	0	1	0	1
	SADMO	0	0	0	1	1	0	0	0	0	0	0	0	0	0	0
	GWH	1	0	0	0	0	0	1	0	0	0	0	0	0	0	0
GDM	Core Hamiltonian	1	0	1	X	0	0	1	0	0	0	0	0	1	0	0
	**SAD**	1	0	1	1	0	0	1	1	X	0	0	0	X	0	1
	AUTOSAD	0	1	0	1	0	0	0	1	0	0	0	0	1	X	1

	SADMO	0	0	0	0	X	0	0	0	0	0	0	0	0	X	0
	GWH	1	0	1	1	0	0	0	0	0	1	0	0	1	0	X
L_BGFS	Core Hamiltonian	1	1	1	1	1	1	0	0	0	0	0	0	0	0	0
	**SAD**	1	0	1	1	1	1	1	1	1	0	0	0	1	1	1
	AUTOSAD	0	1	0	1	1	1	0	1	0	0	1	0	1	1	1
	SADMO	0	0	0	0	0	0	0	0	0	0	0	0	0	0	0
	GWH	1	0	1	1	1	1	0	0	0	1	1	1	1	1	1
Newton	Core Hamiltonian	X	X	X	X	X	0	X	X	X	X	X	X	X	X	X
	**SAD**	1	0	1	1	1	0	1	1	1	0	0	0	1	X	1
	AUTOSAD	0	1	0	1	1	X	0	1	0	0	X	0	1	X	1
	SADMO	0	0	0	0	0	1	X	X	X	0	0	0	0	0	0
	GWH	X	X	X	X	X	X	X	X	X	X	X	X	X	X	X
RCA	Core Hamiltonian	0	0	0	1	0	0	1	0	0	0	0	0	0	1	1
	**SAD**	1	0	1	1	1	1	1	1	1	0	0	0	1	1	1
	AUTOSAD	X	X	X	X	X	X	X	X	X	X	X	X	X	X	X
	SADMO	0	0	0	0	0	0	0	0	0	0	0	0	0	0	0
	GWH	1	1	0	1	0	0	0	0	0	0	0	1	0	0	1
Roothaan	Core Hamiltonian	X	X	X	X	X	X	X	X	X	X	X	X	X	X	X
	**SAD**	1	X	X	1	X	X	1	X	X	0	X	X	1	X	X
	AUTOSAD	0	X	X	1	X	X	0	X	X	0	X	X	1	X	X
	SADMO	X	X	X	X	X	X	X	X	X	X	X	X	X	X	X
	GWH	X	X	X	X	X	X	X	X	X	X	X	X	X	X	X
SCF Metadynamics	Core Hamiltonian	0	0	0	0	0	0	0	0	0	0	0	0	0	0	0
	**SAD**	0	0	0	0	0	0	1	0	0	0	0	0	0	0	0
	AUTOSAD	0	0	0	0	0	0	0	0	0	0	0	0	1	0	0
	SADMO	0	0	0	0	0	0	1	0	0	0	0	0	0	0	0
	GWH	0	0	0	0	0	0	0	0	0	0	0	0	0	0	0

Table S4: Summary of percentage of occurrences of state errors for molecules subject to state errors in the Q-Chem 6.0.2 software package.

**Optimization Method** / **Initial Guess**	DIIS	ADIIS	GDM_LS	GDM	L_BFGS	RCA	SCF Metadynamics	**Total**
Core Hamiltonian	46.7	13.3	33.3	28.6	40.0	26.7	0.0	26.1
SAD	73.3	73.3	53.3	46.2	73.3	73.3	6.7	56.9
AUTOSAD	60.0	53.3	33.3	35.7	60.0	X	6.7	42.5
SADMO	40.0	0.0	13.3	0.0	0.0	0.0	6.7	13.1
GWH	53.3	33.3	13.3	35.7	73.3	33.3	0.0	33.6
**Total**	54.7	33.3	29.3	29.4	49.3	33.3	4.0	

5. Additional Benchmark Results

Previous benchmarking studies have suggested that results of calculations using Minnesota functionals are sensitive to the chosen integration grid[51]. To evaluate the impact of integration grid on these functionals' results in our benchmark, the benchmark values for these functionals were recalculated using an unpruned (99, 590) grid (99 radial shells with 590 grid points per shell) (Figure S3). The unpruned grid (UG) results are broadly in line with those obtained using the

pruned SG-3 grid. For all functionals, the difference in thermodynamics MAE between the pruned and unpruned grids were <0.1 kcal/mol. However, there is a substantial improvement in results for the kinetics of the M06-HF-D3 functional (MAE 2.48 kcal/mol vs. 3.01 kcal/mol) when moving to an unpruned grid, suggesting a higher sensitivity for that functional towards the chosen integration grid. The second highest improvement in kinetics was seen for M06L, though the difference was much smaller (MAE 4.59 kcal/mol vs. 4.74 kcal/mol).

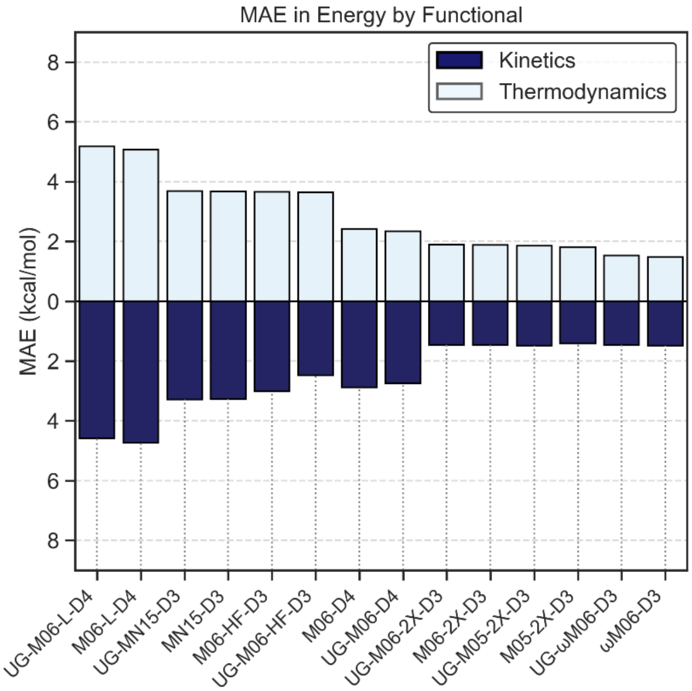

Figure S3: Bar chart of Minnesota functional performance across the TRIP50 dataset comparing results obtained using the default SG-3 grid (no prefix) to those obtained using an unpruned (99, 590) grid (UG prefix). Error in kinetics (dark blue) and thermodynamics (light blue) are reported as mean absolute error (MAE) in kcal/mol compared against reference values computed at DLPNO-CCSD(T)/CBS.

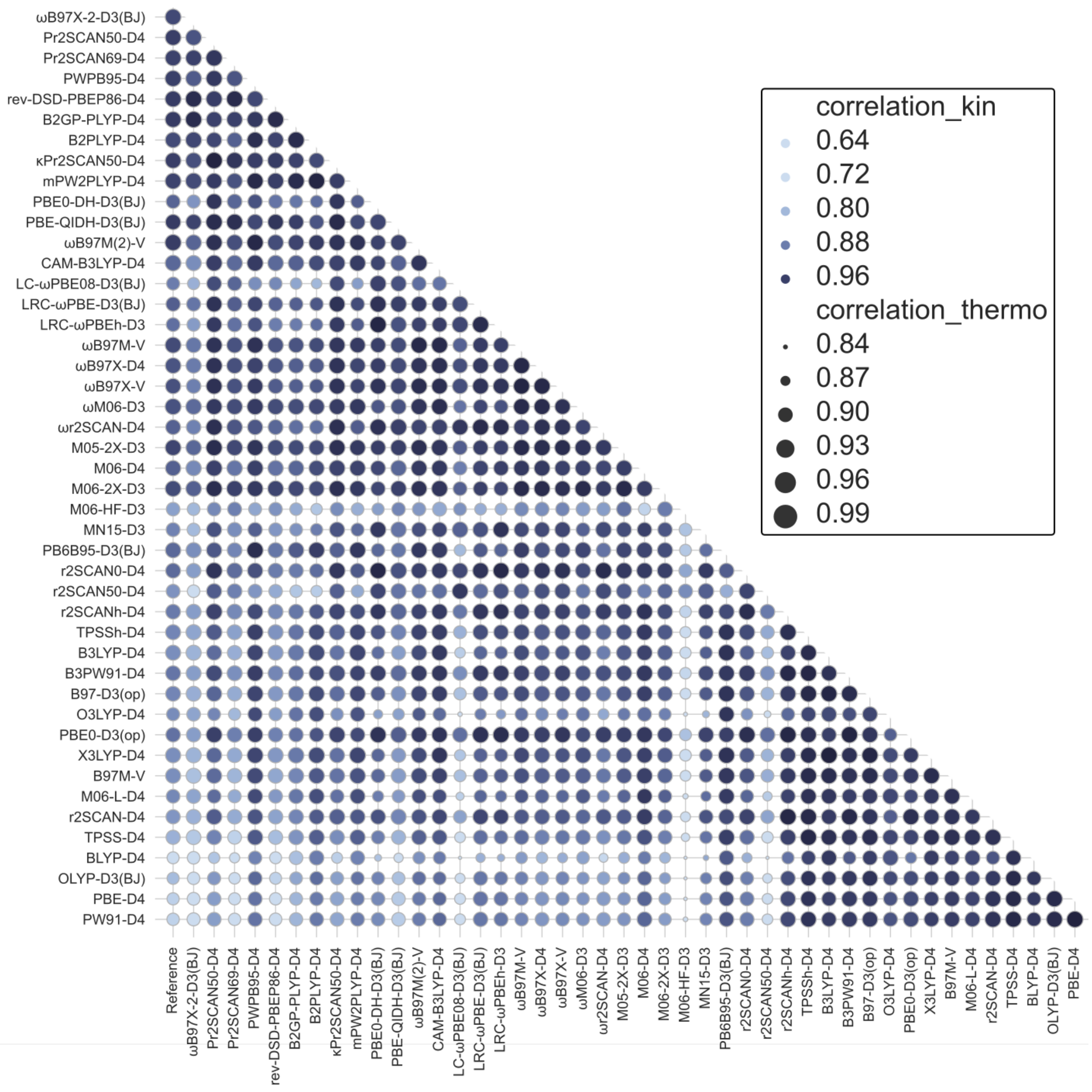

Figure S4: Graphical correlation matrix of functional performance. Pearson correlation coefficients (r) relative to the reference energetics and across functionals are indicated by the color (kinetic values) and size (thermodynamic values) of each point.

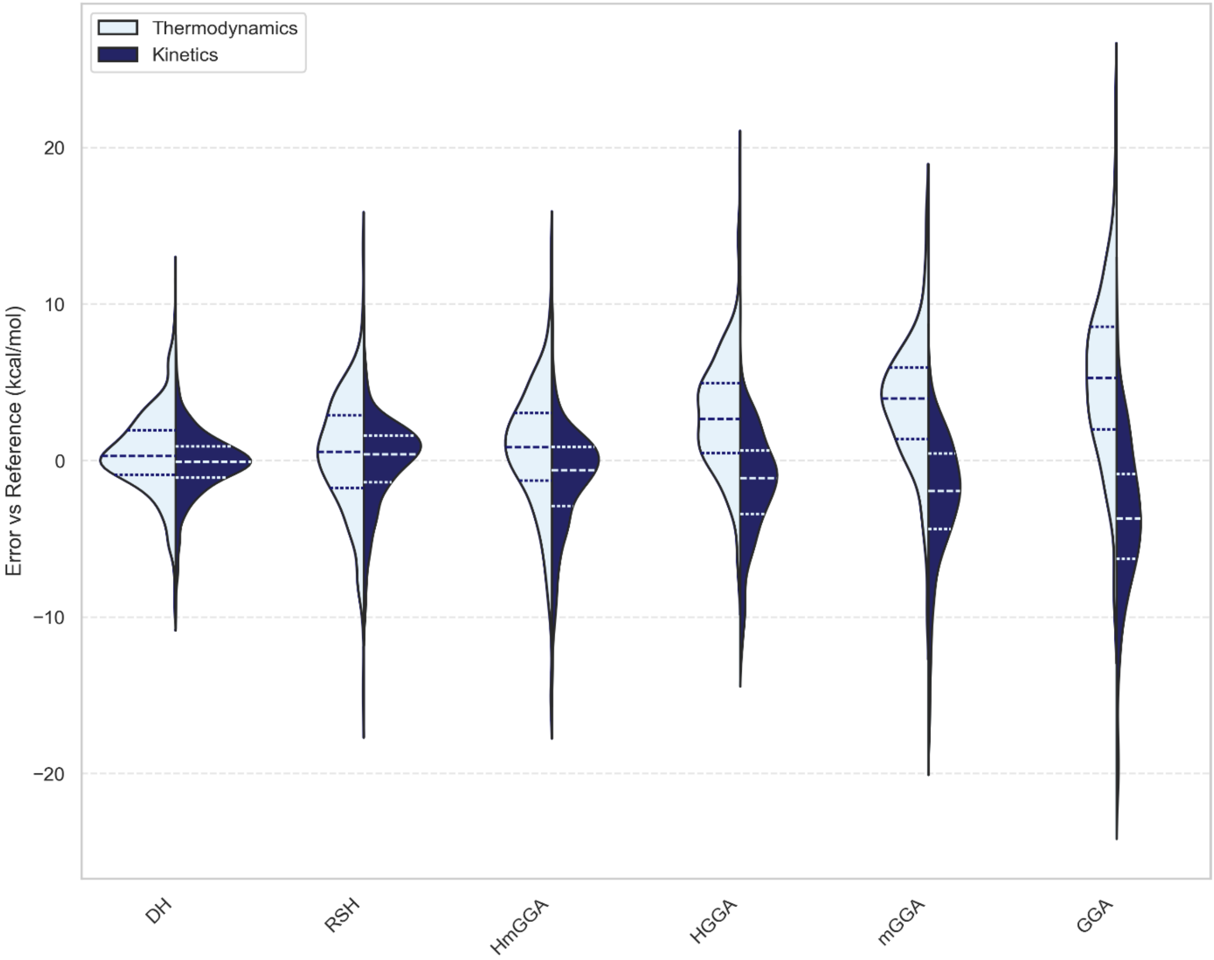

Figure S5: Violin plots of performance for each functional class. Widths correspond to density of data points at a specific error value. Dashed lines on each plot designate the mean and interquartile range (IQR).

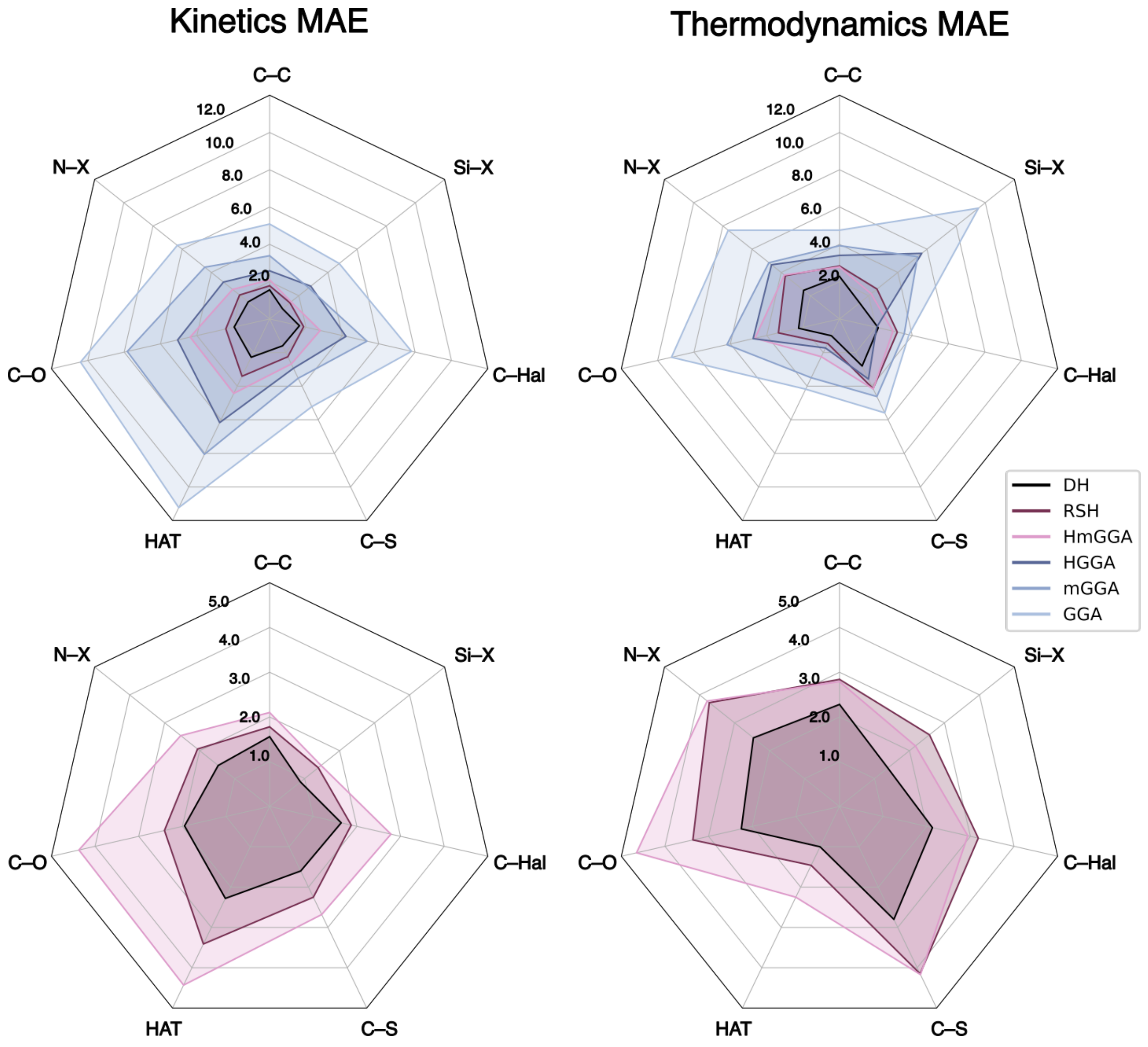

Figure S6: Radar plots of mean absolute error (MAE) for each functional class in kinetics (left) and thermodynamics (right). The data are split into the seven reaction classes. Values in kcal/mol.

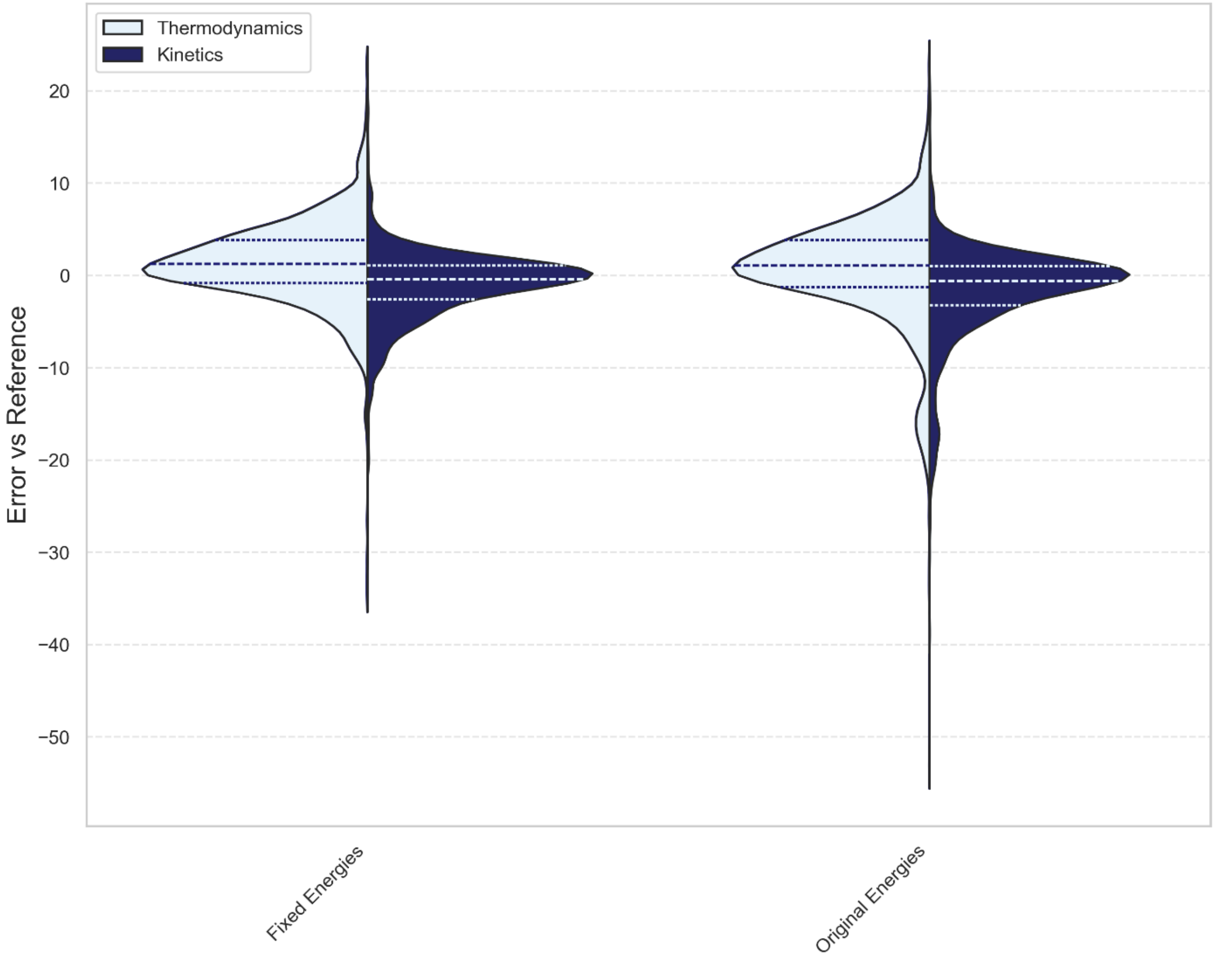

Figure S7: Violin plots of performance for fixed (left) and original (right) SCF convergence. The width of each plot at a given Y value corresponds to the density of data at that value. Dashed lines on each plot designate the mean and interquartile range (IQR).

Table S5: Breakdown of mean absolute error (MAE) for each functional by reaction class. Averages of MAE across a functional class are reported. K, kinetics. T, thermodynamics. Values in kcal/mol.

Theory	Type	C-C MAE K	C-C MAE T	N-X MAE K	N-X MAE T	C-O MAE K	C-O MAE T	HAT MAE K	HAT MAE T	C-S MAE K	C-S MAE T	C-Hal MAE K	C-Hal MAE T	Si-X MAE K	Si-X MAE T
ωB97X-2-D3(BJ)	DH	1.510	2.339	0.966	1.788	1.458	1.673	1.760	1.103	1.221	2.443	0.736	1.472	1.610	0.593
Pr2SCAN50-D4	DH	1.157	1.905	1.334	2.134	0.893	1.442	2.154	0.709	1.863	3.168	1.715	3.431	0.317	0.611
Pr2SCAN69-D4	DH	2.382	1.886	1.610	1.964	3.105	1.989	1.464	1.296	1.702	2.100	1.497	2.946	0.435	0.846
PWPB95-D4	DH	1.360	2.097	1.479	2.515	2.092	1.929	3.263	1.070	1.137	2.043	2.297	0.252	1.553	3.098
rev-DSD-PBEP86-D4	DH	1.661	2.107	1.315	1.998	1.825	0.722	1.311	1.116	1.190	0.930	0.640	1.020	0.445	0.625
B2GP-PLYP-D4	DH	1.296	2.151	1.008	1.820	1.175	1.986	1.633	0.877	0.970	1.890	0.987	0.431	0.322	0.395
B2PLYP-D4	DH	1.920	2.694	1.632	2.163	2.905	3.839	3.331	1.324	1.439	2.495	2.439	0.817	0.976	1.952
κPr2SCAN50-D4	DH	1.172	1.921	1.323	2.019	1.148	2.107	2.218	0.619	1.895	3.336	1.786	3.572	0.396	0.479
mPW2PLYP-D4	DH	1.488	2.373	1.241	2.070	1.759	2.381	2.856	0.816	1.210	2.386	1.976	0.662	0.667	1.315
PBE0-DH-D3(BJ)	DH	2.133	4.045	3.004	5.959	2.459	4.857	3.276	0.994	3.815	7.631	3.094	6.189	1.641	3.282
PBE-QIDH-D3(BJ)	DH	1.935	2.759	1.774	3.477	2.497	3.385	1.263	0.865	1.965	3.924	2.069	4.138	0.733	0.508
ωB97M(2)-V	DH	0.660	1.140	0.910	1.622	2.100	0.647	2.845	1.225	0.805	1.295	0.429	0.658	1.429	2.859
CAM-B3LYP-D4	RSH	1.264	1.947	1.857	2.971	1.816	1.084	4.027	1.528	0.997	1.903	1.405	0.355	1.492	1.774
LC-ωPBE08-D3(BJ)	RSH	3.848	6.265	3.009	5.600	5.547	10.421	1.715	2.227	4.105	8.210	3.712	5.430	0.375	0.519
LRC-ωPBE-D3(BJ)	RSH	1.710	3.134	2.815	5.530	1.797	2.418	4.572	1.175	2.596	5.111	2.564	5.127	2.388	4.776
LRC-ωPBEh-D3	RSH	2.130	4.117	3.441	6.519	2.842	4.231	5.038	0.888	3.224	6.057	2.763	5.525	2.200	4.400
ωB97M-V	RSH	0.758	1.210	1.064	1.871	1.702	1.219	2.817	1.854	1.114	2.134	1.018	1.602	1.774	3.548
ωB97X-D4	RSH	1.800	2.302	1.543	2.247	1.770	2.228	2.953	1.443	2.222	3.169	1.277	2.555	1.348	2.696
ωB97X-V	RSH	1.582	2.072	1.939	3.609	2.164	2.519	3.007	1.637	1.904	3.329	1.389	2.778	1.912	3.824
ωM06-D3	RSH	1.377	1.960	0.839	1.278	1.546	1.198	3.268	1.269	1.404	2.337	0.473	0.790	0.794	1.219
ωr2SCAN-D4	RSH	1.596	2.598	2.005	3.887	2.486	4.965	3.255	1.142	2.644	5.094	2.230	4.460	0.207	0.366
M05-2X-D3	HmGGA	1.103	1.573	0.962	1.716	1.963	3.464	2.383	1.850	1.488	2.961	2.072	1.744	0.531	0.538
M06-D4	HmGGA	2.169	2.527	2.779	3.183	4.442	2.042	4.711	1.312	2.453	3.082	2.754	2.990	1.090	2.180
M06-2X-D3	HmGGA	1.298	2.077	1.057	1.626	1.844	2.660	2.885	1.747	1.348	2.697	0.961	1.875	0.556	1.006
M06-HF-D3	HmGGA	1.739	1.988	2.500	3.539	7.953	9.156	4.179	4.885	2.386	3.011	1.348	1.691	2.145	2.660
MN15-D3	HmGGA	2.638	3.297	3.608	6.927	4.563	7.149	5.144	1.662	3.072	4.783	2.925	1.800	0.925	0.664
PB6B95-D3(BJ)	HmGGA	2.360	3.983	2.221	3.708	3.844	4.529	4.792	1.800	2.132	3.227	3.855	0.367	2.445	4.890
r2SCAN0-D4	HmGGA	1.623	2.644	2.215	3.790	2.772	2.987	3.664	1.342	3.176	5.698	2.555	5.110	0.725	1.093
r2SCAN50-D4	HmGGA	2.448	3.820	2.383	3.827	5.333	8.404	2.410	3.740	3.669	7.338	2.450	4.657	2.385	0.866
r2SCANh-D4	HmGGA	2.193	2.657	3.451	4.657	5.024	1.925	6.372	1.191	3.437	4.759	3.526	5.492	1.367	2.733
TPSSh-D4	HmGGA	3.414	3.373	4.228	4.812	5.973	4.190	7.753	2.968	3.616	4.161	5.375	3.626	2.531	5.062
B3LYP-D4	HGGA	2.888	3.758	3.078	3.603	5.565	5.050	6.324	1.309	3.218	3.094	4.898	0.660	2.345	4.690
B3PW91-D4	HGGA	2.320	2.499	3.362	5.178	4.187	2.155	6.293	1.261	2.934	3.501	3.669	3.509	2.575	5.149
B97-D3(op)	HGGA	3.000	2.835	3.338	4.328	5.554	5.024	6.637	2.341	3.668	3.938	5.421	2.299	3.684	7.368
O3LYP-D4	HGGA	2.701	4.927	3.023	5.372	7.197	10.967	5.880	3.237	1.852	3.477	3.479	0.749	3.590	7.181
PBE0-D3(op)	HGGA	1.906	2.805	3.270	5.851	2.810	0.998	5.732	1.058	2.829	4.254	2.873	4.741	2.466	4.932
X3LYP-D4	HGGA	2.662	3.632	2.933	3.614	5.065	4.318	6.182	1.175	3.124	3.231	4.877	0.294	2.245	4.491
B97M-V	mGGA	2.345	3.193	3.188	3.738	6.747	5.191	6.243	2.854	2.222	3.648	4.609	1.921	2.534	5.069
M06-L-D4	mGGA	3.226	5.251	4.171	4.475	8.606	8.488	7.628	4.614	2.979	3.898	4.319	2.804	2.818	5.637
r2SCAN-D4	mGGA	3.084	2.892	4.698	5.301	7.179	3.754	8.369	2.170	3.983	5.595	5.081	5.804	2.021	4.041
TPSS-D4	mGGA	4.913	4.377	5.725	5.852	8.804	7.327	10.010	4.355	4.936	5.327	7.409	3.700	3.351	6.702
BLYP-D4	GGA	5.851	6.145	5.972	5.775	11.622	12.194	10.678	3.888	6.060	5.270	8.700	1.169	4.235	8.470
OLYP-D3(BJ)	GGA	4.885	3.991	5.946	8.149	10.164	10.135	10.379	5.163	4.718	4.951	6.791	3.517	5.820	11.639
PBE-D4	GGA	4.804	4.436	6.708	8.390	9.921	7.382	11.882	3.934	5.017	5.981	7.745	5.345	4.690	9.381
PW91-D4	GGA	4.831	4.459	6.698	8.204	9.887	7.242	11.965	3.725	5.086	6.149	7.985	5.251	4.320	8.641
HF-D4	ab initio	4.777	4.147	8.498	9.189	12.859	15.164	11.349	10.867	3.074	5.195	8.173	7.372	12.124	5.173
**Average**	**DH**	**1.556**	**2.285**	**1.466**	**2.461**	**1.951**	**2.246**	**2.281**	**1.001**	**1.601**	**2.803**	**1.639**	**2.132**	**0.877**	**1.380**
**Average**	**GGA**	**5.093**	**4.758**	**6.331**	**7.630**	**10.399**	**9.238**	**11.226**	**4.177**	**5.220**	**5.588**	**7.805**	**3.820**	**4.766**	**9.533**
**Average**	**HGGA**	**2.580**	**3.409**	**3.168**	**4.658**	**5.063**	**4.752**	**6.175**	**1.730**	**2.937**	**3.583**	**4.203**	**2.042**	**2.818**	**5.635**
**Average**	**HmGGA**	**2.099**	**2.794**	**2.540**	**3.779**	**4.371**	**4.651**	**4.429**	**2.250**	**2.678**	**4.172**	**2.782**	**2.935**	**1.470**	**2.169**
**Average**	**RSH**	**1.785**	**2.845**	**2.057**	**3.723**	**2.408**	**3.365**	**3.406**	**1.463**	**2.246**	**4.149**	**1.870**	**3.180**	**1.388**	**2.569**
**Average**	**mGGA**	**3.392**	**3.928**	**4.445**	**4.842**	**7.834**	**6.190**	**8.062**	**3.498**	**3.530**	**4.617**	**5.354**	**3.557**	**2.681**	**5.362**

6. References

(1) Chai, J.-D.; Head-Gordon, M. Long-Range Corrected Double-Hybrid Density Functionals. *J. Chem. Phys.* **2009**, *131* (17), 174105. https://doi.org/10.1063/1.3244209.

(2) Wittmann, L.; Neugebauer, H.; Grimme, S.; Bursch, M. Dispersion-Corrected r2SCAN Based Double-Hybrid Functionals. *J. Chem. Phys.* **2023**, *159* (22), 224103. https://doi.org/10.1063/5.0174988.

(3) Goerigk, L.; Grimme, S. Efficient and Accurate Double-Hybrid-Meta-GGA Density Functionals—Evaluation with the Extended GMTKN30 Database for General Main Group Thermochemistry, Kinetics, and Noncovalent Interactions. *J. Chem. Theory Comput.* **2011**, *7* (2), 291–309. https://doi.org/10.1021/ct100466k.

(4) Santra, G.; Sylvetsky, N.; Martin, J. M. L. Minimally Empirical Double-Hybrid Functionals Trained against the GMTKN55 Database: revDSD-PBEP86-D4, revDOD-PBE-D4, and DOD-SCAN-D4. *J. Phys. Chem. A* **2019**, *123* (24), 5129–5143. https://doi.org/10.1021/acs.jpca.9b03157.

(5) Karton, A.; Tarnopolsky, A.; Lamère, J.-F.; Schatz, G. C.; Martin, J. M. L. Highly Accurate First-Principles Benchmark Data Sets for the Parametrization and Validation of Density Functional and Other Approximate Methods. Derivation of a Robust, Generally Applicable, Double-Hybrid Functional for Thermochemistry and Thermochemical Kinetics. *J. Phys. Chem. A* **2008**, *112* (50), 12868–12886. https://doi.org/10.1021/jp801805p.

(6) Grimme, S. Semiempirical Hybrid Density Functional with Perturbative Second-Order Correlation. *J. Chem. Phys.* **2006**, *124* (3), 034108. https://doi.org/10.1063/1.2148954.

(7) Schwabe, T.; Grimme, S. Towards Chemical Accuracy for the Thermodynamics of Large Molecules: New Hybrid Density Functionals Including Non-Local Correlation Effects. *Phys. Chem. Chem. Phys.* **2006**, *8* (38), 4398–4401. https://doi.org/10.1039/B608478H.

(8) Brémond, E.; Adamo, C. Seeking for Parameter-Free Double-Hybrid Functionals: The PBE0-DH Model. *J. Chem. Phys.* **2011**, *135* (2), 024106. https://doi.org/10.1063/1.3604569.

(9) Brémond, É.; Sancho-García, J. C.; Pérez-Jiménez, Á. J.; Adamo, C. Communication: Double-Hybrid Functionals from Adiabatic-Connection: The QIDH Model. *J. Chem. Phys.* **2014**, *141* (3), 031101. https://doi.org/10.1063/1.4890314.

(10) Mardirossian, N.; Head-Gordon, M. Survival of the Most Transferable at the Top of Jacob's Ladder: Defining and Testing the ωB97M(2) Double Hybrid Density Functional. *J. Chem. Phys.* **2018**, *148* (24), 241736. https://doi.org/10.1063/1.5025226.

(11) Yanai, T.; Tew, D. P.; Handy, N. C. A New Hybrid Exchange–Correlation Functional Using the Coulomb-Attenuating Method (CAM-B3LYP). *Chem. Phys. Lett.* **2004**, *393* (1), 51–57. https://doi.org/10.1016/j.cplett.2004.06.011.

(12) Weintraub, E.; Henderson, T. M.; Scuseria, G. E. Long-Range-Corrected Hybrids Based on a New Model Exchange Hole. *J. Chem. Theory Comput.* **2009**, *5* (4), 754–762. https://doi.org/10.1021/ct800530u.

(13) Rohrdanz, M. A.; Herbert, J. M. Simultaneous Benchmarking of Ground- and Excited-State Properties with Long-Range-Corrected Density Functional Theory. *J. Chem. Phys.* **2008**, *129* (3), 034107. https://doi.org/10.1063/1.2954017.

(14) Rohrdanz, M. A.; Martins, K. M.; Herbert, J. M. A Long-Range-Corrected Density Functional That Performs Well for Both Ground-State Properties and Time-Dependent Density Functional Theory Excitation Energies, Including Charge-Transfer Excited States. *J. Chem. Phys.* **2009**, *130* (5), 054112. https://doi.org/10.1063/1.3073302.

(15) *ωB97M-V: A combinatorially optimized, range-separated hybrid, meta-GGA density functional with VV10 nonlocal correlation | The Journal of Chemical Physics | AIP Publishing*. https://pubs.aip.org/aip/jcp/article/144/21/214110/313155/B97M-V-A-combinatorially-optimized-range-separated (accessed 2025-06-27).

(16) Mardirossian, N.; Head-Gordon, M. ωB97X-V: A 10-Parameter, Range-Separated Hybrid, Generalized Gradient Approximation Density Functional with Nonlocal Correlation, Designed by a Survival-of-the-Fittest Strategy. *Phys. Chem. Chem. Phys.* **2014**, *16* (21), 9904–9924. https://doi.org/10.1039/C3CP54374A.

(17) Lin, Y.-S.; Li, G.-D.; Mao, S.-P.; Chai, J.-D. Long-Range Corrected Hybrid Density Functionals with Improved Dispersion Corrections. *J. Chem. Theory Comput.* **2013**, *9* (1), 263–272. https://doi.org/10.1021/ct300715s.

(18) Zhao, Y.; Schultz, N. E.; Truhlar, D. G. Design of Density Functionals by Combining the Method of Constraint Satisfaction with Parametrization for Thermochemistry, Thermochemical Kinetics, and Noncovalent Interactions. *J. Chem. Theory Comput.* **2006**, *2* (2), 364–382. https://doi.org/10.1021/ct0502763.

(19) Zhao, Y.; Truhlar, D. G. The M06 Suite of Density Functionals for Main Group Thermochemistry, Thermochemical Kinetics, Noncovalent Interactions, Excited States, and Transition Elements: Two New Functionals and Systematic Testing of Four M06-Class Functionals and 12 Other Functionals. *Theor. Chem. Acc.* **2008**, *120* (1), 215–241. https://doi.org/10.1007/s00214-007-0310-x.

(20) Zhao, Y.; Truhlar, D. G. Density Functional for Spectroscopy: No Long-Range Self-Interaction Error, Good Performance for Rydberg and Charge-Transfer States, and Better Performance on Average than B3LYP for Ground States. *J. Phys. Chem. A* **2006**, *110* (49), 13126–13130. https://doi.org/10.1021/jp066479k.

(21) Yu, H. S.; He, X.; Li, S. L.; Truhlar, D. G. MN15: A Kohn–Sham Global-Hybrid Exchange–Correlation Density Functional with Broad Accuracy for Multi-Reference and Single-Reference Systems and Noncovalent Interactions. *Chem. Sci.* **2016**, *7* (8), 5032–5051. https://doi.org/10.1039/C6SC00705H.

(22) Zhao, Y.; Truhlar, D. G. Design of Density Functionals That Are Broadly Accurate for Thermochemistry, Thermochemical Kinetics, and Nonbonded Interactions. *J. Phys. Chem. A* **2005**, *109* (25), 5656–5667. https://doi.org/10.1021/jp050536c.

(23) Bursch, M.; Neugebauer, H.; Ehlert, S.; Grimme, S. Dispersion Corrected r2SCAN Based Global Hybrid Functionals: r2SCANh, r2SCAN0, and r2SCAN50. *J. Chem. Phys.* **2022**, *156* (13), 134105. https://doi.org/10.1063/5.0086040.

(24) Staroverov, V. N.; Scuseria, G. E.; Tao, J.; Perdew, J. P. Comparative Assessment of a New Nonempirical Density Functional: Molecules and Hydrogen-Bonded Complexes. *J. Chem. Phys.* **2003**, *119* (23), 12129–12137. https://doi.org/10.1063/1.1626543.

(25) Becke, A. D. Density-functional Thermochemistry. III. The Role of Exact Exchange. *J. Chem. Phys.* **1993**, *98* (7), 5648–5652. https://doi.org/10.1063/1.464913.

(26) Stephens, P. J.; Devlin, F. J.; Chabalowski, C. F.; Frisch, M. J. Ab Initio Calculation of Vibrational Absorption and Circular Dichroism Spectra Using Density Functional Force Fields. *J. Phys. Chem.* **1994**, *98* (45), 11623–11627. https://doi.org/10.1021/j100096a001.

(27) Becke, A. D. Density-Functional Thermochemistry. V. Systematic Optimization of Exchange-Correlation Functionals. *J. Chem. Phys.* **1997**, *107* (20), 8554–8560. https://doi.org/10.1063/1.475007.

(28) Hoe, W.-M.; Cohen, A. J.; Handy, N. C. Assessment of a New Local Exchange Functional OPTX. *Chem. Phys. Lett.* **2001**, *341* (3), 319–328. https://doi.org/10.1016/S0009-2614(01)00581-4.

(29) Adamo, C.; Barone, V. Toward Reliable Density Functional Methods without Adjustable Parameters: The PBE0 Model. *J. Chem. Phys.* **1999**, *110* (13), 6158–6170. https://doi.org/10.1063/1.478522.

(30) Xu, X.; Goddard, W. A. The X3LYP Extended Density Functional for Accurate Descriptions of Nonbond Interactions, Spin States, and Thermochemical Properties. *Proc. Natl. Acad. Sci.* **2004**, *101* (9), 2673–2677. https://doi.org/10.1073/pnas.0308730100.

(31) Mardirossian, N.; Head-Gordon, M. Mapping the Genome of Meta-Generalized Gradient Approximation Density Functionals: The Search for B97M-V. *J. Chem. Phys.* **2015**, *142* (7), 074111. https://doi.org/10.1063/1.4907719.

(32) Zhao, Y.; Truhlar, D. G. A New Local Density Functional for Main-Group Thermochemistry, Transition Metal Bonding, Thermochemical Kinetics, and Noncovalent Interactions. *J. Chem. Phys.* **2006**, *125* (19), 194101. https://doi.org/10.1063/1.2370993.

(33) Furness, J. W.; Kaplan, A. D.; Ning, J.; Perdew, J. P.; Sun, J. Accurate and Numerically Efficient r2SCAN Meta-Generalized Gradient Approximation. *J. Phys. Chem. Lett.* **2020**, *11* (19), 8208–8215. https://doi.org/10.1021/acs.jpclett.0c02405.

(34) Tao, J.; Perdew, J. P.; Staroverov, V. N.; Scuseria, G. E. Climbing the Density Functional Ladder: Nonempirical Meta--Generalized Gradient Approximation Designed for Molecules and Solids. *Phys. Rev. Lett.* **2003**, *91* (14), 146401. https://doi.org/10.1103/PhysRevLett.91.146401.

(35) Becke, A. D. Density-Functional Exchange-Energy Approximation with Correct Asymptotic Behavior. *Phys. Rev. A* **1988**, *38* (6), 3098–3100. https://doi.org/10.1103/PhysRevA.38.3098.

(36) Lee, C.; Yang, W.; Parr, R. G. Development of the Colle-Salvetti Correlation-Energy Formula into a Functional of the Electron Density. *Phys. Rev. B* **1988**, *37* (2), 785–789. https://doi.org/10.1103/PhysRevB.37.785.

(37) Miehlich, B.; Savin, A.; Stoll, H.; Preuss, H. Results Obtained with the Correlation Energy Density Functionals of Becke and Lee, Yang and Parr. *Chem. Phys. Lett.* **1989**, *157* (3), 200–206. https://doi.org/10.1016/0009-2614(89)87234-3.

(38) HANDY, N. C.; and COHEN, A. J. Left-Right Correlation Energy. *Mol. Phys.* **2001**, *99* (5), 403–412. https://doi.org/10.1080/00268970010018431.

(39) Perdew, J. P.; Burke, K.; Ernzerhof, M. Generalized Gradient Approximation Made Simple. *Phys. Rev. Lett.* **1996**, *77* (18), 3865–3868. https://doi.org/10.1103/PhysRevLett.77.3865.

(40) Perdew, J. P.; Chevary, J. A.; Vosko, S. H.; Jackson, K. A.; Pederson, M. R.; Singh, D. J.; Fiolhais, C. Atoms, Molecules, Solids, and Surfaces: Applications of the Generalized Gradient Approximation for Exchange and Correlation. *Phys. Rev. B* **1992**, *46* (11), 6671–6687. https://doi.org/10.1103/PhysRevB.46.6671.

(41) Hartree, D. R. The Wave Mechanics of an Atom with a Non-Coulomb Central Field. Part I. Theory and Methods. *Math. Proc. Camb. Philos. Soc.* **1928**, *24* (1), 89–110. https://doi.org/10.1017/S0305004100011919.

(42) Fock, V. Näherungsmethode zur Lösung des quantenmechanischen Mehrkörperproblems. *Z. Für Phys.* **1930**, *61* (1), 126–148. https://doi.org/10.1007/BF01340294.

(43) *RDKit: open-source cheminformatics.* https://www.rdkit.org.

(44) Gaussian 16, Revision C.01, M. J. Frisch, G. W. Trucks, H. B. Schlegel, G. E. Scuseria, M. A. Robb, J. R. Cheeseman, G. Scalmani, V. Barone, G. A. Petersson, H. Nakatsuji, X. Li, M. Caricato, A. V. Marenich, J. Bloino, B. G. Janesko, R. Gomperts, B. Mennucci, H. P. Hratchian, J. V. Ortiz, A. F. Izmaylov, J. L. Sonnenberg, D. Williams-Young, F. Ding, F. Lipparini, F. Egidi, J. Goings, B. Peng, A. Petrone, T. Henderson, D. Ranasinghe, V. G. Zakrzewski, J. Gao, N. Rega, G. Zheng, W. Liang, M. Hada, M. Ehara, K. Toyota, R. Fukuda, J. Hasegawa, M. Ishida, T. Nakajima, Y. Honda, O. Kitao, H. Nakai, T. Vreven, K. Throssell, J. A. Jr. S-96 Montgomery, J. E. Peralta, F. Ogliaro, M. J. Bearpark, J. J. Heyd, E. N. Brothers, K. N. Kudin, V. N. Staroverov, T. A. Keith, R. Kobayashi, J. Normand, K. Raghavachari, A. P. Rendell, J. C. Burant, S. S. Iyengar, J. Tomasi, M. Cossi, J. M. Millam, M. Klene, C. Adamo, R. Cammi, J. W. Ochterski, R. L. Martin, K. Morokuma, O. Farkas, J. B. Foresman, D. J. Fox, Gaussian, Inc., Wallingford CT, **2016**.

(45) Chai, J.-D.; Head-Gordon, M. Long-Range Corrected Hybrid Density Functionals with Damped Atom–Atom Dispersion Corrections. *Phys. Chem. Chem. Phys.* **2008**, *10* (44), 6615–6620. https://doi.org/10.1039/B810189B.

(46) Weigend, F.; Ahlrichs, R. Balanced Basis Sets of Split Valence, Triple Zeta Valence and Quadruple Zeta Valence Quality for H to Rn: Design and Assessment of Accuracy. *Phys. Chem. Chem. Phys.* **2005**, *7* (18), 3297–3305. https://doi.org/10.1039/B508541A.

(47) de Souza, B. GOAT: A Global Optimization Algorithm for Molecules and Atomic Clusters. *Angew. Chem. Int. Ed.* **2025**, *64* (18), e202500393. https://doi.org/10.1002/anie.202500393.

(48) Bannwarth, C.; Ehlert, S.; Grimme, S. GFN2-xTB—An Accurate and Broadly Parametrized Self-Consistent Tight-Binding Quantum Chemical Method with Multipole Electrostatics and Density-Dependent Dispersion Contributions. *J. Chem. Theory Comput.* **2019**, *15* (3), 1652–1671. https://doi.org/10.1021/acs.jctc.8b01176.

(49) Neese, F. Software Update: The ORCA Program System—Version 5.0. *WIREs Comput. Mol. Sci.* **2022**, *12* (5), e1606. https://doi.org/10.1002/wcms.1606.

(50) Thom, A. J. W.; Head-Gordon, M. Locating Multiple Self-Consistent Field Solutions: An Approach Inspired by Metadynamics. *Phys. Rev. Lett.* **2008**, *101* (19), 193001. https://doi.org/10.1103/PhysRevLett.101.193001.

(51) Mardirossian, N.; Head-Gordon, M. How Accurate Are the Minnesota Density Functionals for Noncovalent Interactions, Isomerization Energies, Thermochemistry, and Barrier Heights Involving Molecules Composed of Main-Group Elements? *J. Chem. Theory Comput.* **2016**, *12* (9), 4303–4325. https://doi.org/10.1021/acs.jctc.6b00637.
